# Identification of a Functional Connectome for Long-Term Fear Memory in Mice

**DOI:** 10.1371/journal.pcbi.1002853

**Published:** 2013-01-03

**Authors:** Anne L. Wheeler, Cátia M. Teixeira, Afra H. Wang, Xuejian Xiong, Natasa Kovacevic, Jason P. Lerch, Anthony R. McIntosh, John Parkinson, Paul W. Frankland

**Affiliations:** 1 Program in Neurosciences and Mental Health, The Hospital for Sick Children, Toronto, Canada; 2 Institute of Medical Science, University of Toronto, Toronto, Canada; 3 Program in Molecular Structure and Function, The Hospital for Sick Children, Toronto, Canada; 4 Rotman Research Institute, Baycrest Centre, Toronto, Canada; 5 Department of Medical Biophysics, University of Toronto, Toronto, Canada; 6 Department of Psychology, University of Toronto, Toronto, Canada; 7 Departments of Biochemistry and Molecular Genetics, University of Toronto, Toronto, Canada; 8 Department of Physiology, University of Toronto, Toronto, Canada; Indiana University, United States of America

## Abstract

Long-term memories are thought to depend upon the coordinated activation of a broad network of cortical and subcortical brain regions. However, the distributed nature of this representation has made it challenging to define the neural elements of the memory trace, and lesion and electrophysiological approaches provide only a narrow window into what is appreciated a much more global network. Here we used a global mapping approach to identify networks of brain regions activated following recall of long-term fear memories in mice. Analysis of Fos expression across 84 brain regions allowed us to identify regions that were co-active following memory recall. These analyses revealed that the functional organization of long-term fear memories depends on memory age and is altered in mutant mice that exhibit premature forgetting. Most importantly, these analyses indicate that long-term memory recall engages a network that has a distinct thalamic-hippocampal-cortical signature. This network is concurrently integrated and segregated and therefore has small-world properties, and contains hub-like regions in the prefrontal cortex and thalamus that may play privileged roles in memory expression.

## Author Summary

Memory retrieval is thought to involve the coordinated activation of multiple regions of the brain, rather than localized activity in a specific region. In order to visualize networks of brain regions activated by recall of a fear memory in mice, we quantified expression of an activity-regulated gene (c-fos) that is induced by neural activity. This allowed us to identify collections of brain regions where Fos expression co-varies across mice, and presumably form components of a network that are co-active during recall of long-term fear memory. This analysis suggested that expression of a long-term fear memory is an emergent property of large scale neural network interactions. This network has a distinct thalamic-hippocampal-cortical signature and, like many real-world networks as well as other anatomical and functional brain networks, has small-world architecture with a subset of highly-connected hub nodes that may play more central roles in memory expression.

## Introduction

Long-term memories are thought to be represented by changes in the strength of connections among neurons in the brain [1], [2]. While much is understood about the molecular, cellular and structural changes that contribute to the changes in connection strength between neurons [3], [4], [5], it has been challenging to precisely define which subsets of neurons constitute the memory trace for at least two reasons. First, long-term memories are thought to be distributed, and depend on the collective activity of groups of neurons (or cell assemblies [6]) across a broad network of cortical and subcortical brain regions [7]. Second, memory expression likely depends upon network-wide, coordinated activation of these cell assemblies, rather than an overall, net increase in network activity [8], [9]. Electrophysiological approaches have been useful in linking regional activity and coordination of inter-regional activity to memory processing [10]. However, they necessarily provide only a narrow window into what is appreciated to be a much more global network.

**Figure 1:**
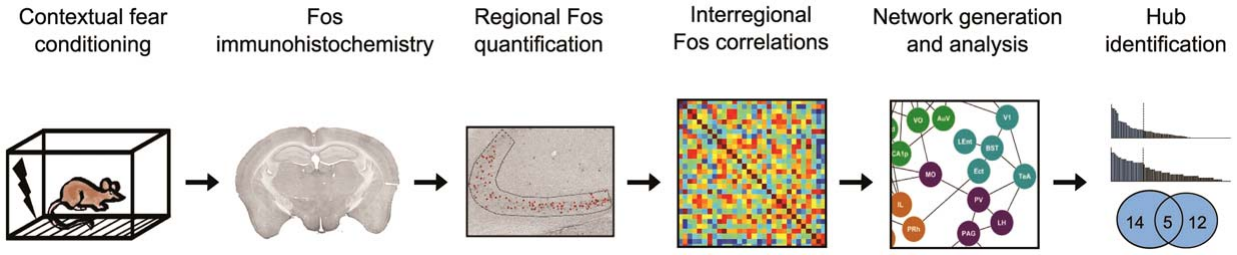
Overview of experimental approach. Mice were fear conditioned, and fear memory was tested after either a short- or long retention delay. In order to identify neurons activated by memory recall, following testing brain sections were stained for the activity-dependent gene *c-fos*. Fos expression was subsequently quantified in 84 brain regions, and a complete set of inter-regional correlations computed in order to identify collections of brain regions where Fos expression co-varies across mice. The most robust correlations were then used to generate functional networks for long-term fear memory, and network properties and hubs were characterized using graph theoretical approaches.

Imaging-based approaches can detect coordinated activity across distributed and spatially remote brain regions, and therefore have been useful in defining functional networks (see **Supplementary Note in Text S1**). Here we have developed a brain-wide imaging approach to study the network organization of long-term contextual fear memories in mice (**Figure 1**). Sustained neural activity leads to the induction of activity-regulated genes such as *c-fos* [11], [12]. As Fos protein may be resolved at the level of the nucleus, immunohistochemical approaches may be used to generate high resolution, brain-wide maps of Fos expression induced by memory recall. Subsequent computation of inter-regional correlations allows us to identify collections of brain regions where Fos expression co-varies across mice, and presumably form components of a network that are co-active during recall of long-term fear memory. This analysis of functional connectivity suggests that expression of a long-term fear memory is an emergent property of large scale neural network interactions. This network has a distinct thalamic-hippocampal-cortical signature and, like many real-world networks [13] as well as other anatomical and functional brain networks [14], has small-world architecture with a subset of highly-connected hub nodes that may play more central roles in memory expression.

## Results

### Fos expression is induced by contextual fear memory recall

**Figure 2:**
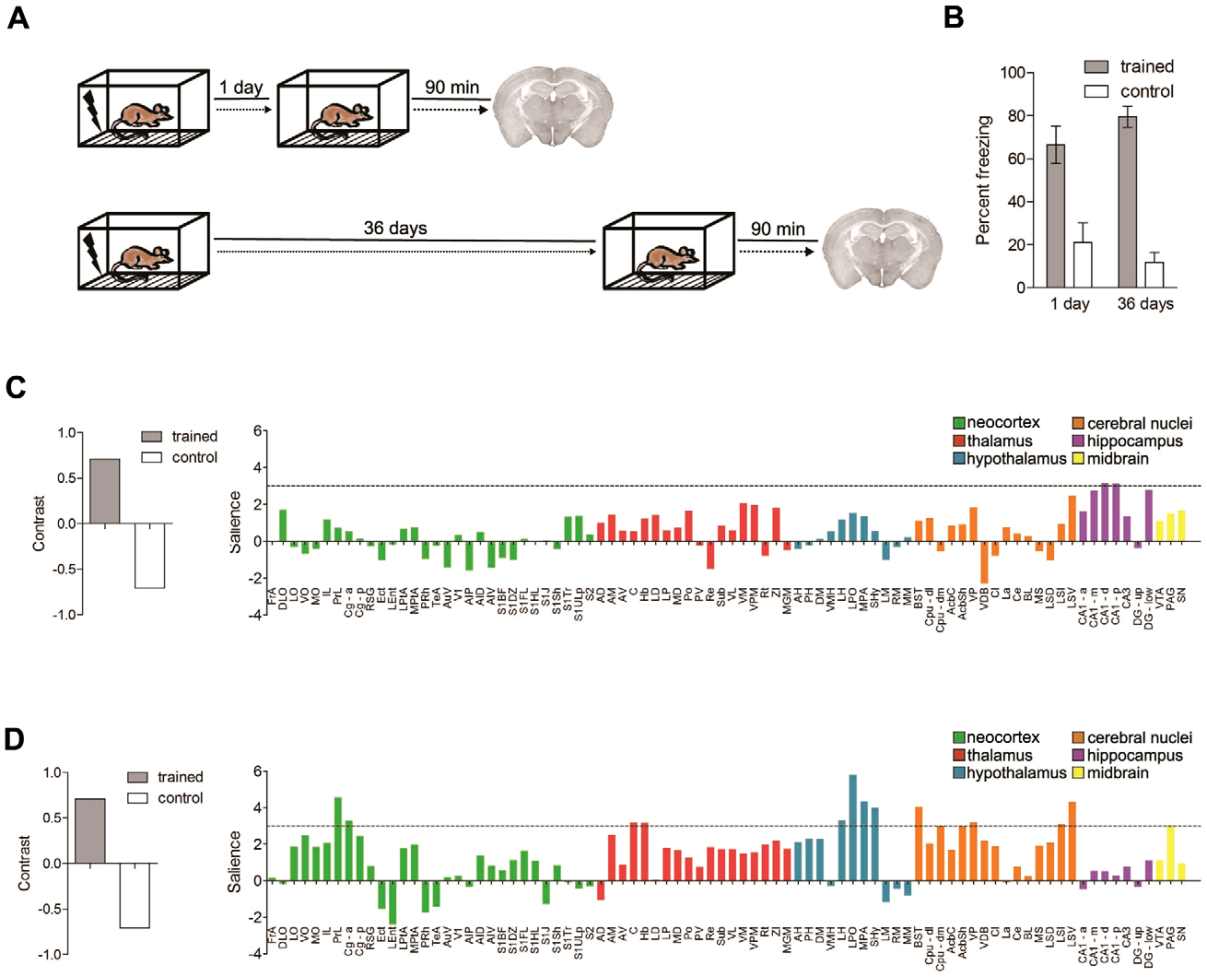
Fos is induced by contextual fear memory recall. **A.** Experimental design. Mice were trained, and fear memory was assessed either 1 (short delay) or 36 (long delay) days later. Ninety minutes following this test, brains were removed and expression of the activity-regulated gene, c-fos, was evaluated immunohistochemically. **B.** Percent freezing at the 1 day or 36 day retention test in trained (black bars) or control (open bars) mice. **C–D.** Task PLS analysis of Fos expression in trained vs. control mice tested 1 or 36 days following training. These analyses identified LVs (left graph) that strongly differentiated the trained vs. control conditions at both the (**C**) short and (**D**) long retention delays. Salience scores (right) identify regions that maximally differentiate between these conditions at both the (**C**) short and (**D**) long retention delays. The hatched line reflects a salience score of 3, above which the contribution of the regions is considered reliable. At the short delay, Fos expression in the hippocampus contributed strongly to this contrast, whereas at the longer retention delay, Fos expression in multiple brain regions contributed to the contrast.

In order to characterize regional activation following long-term memory recall, we used a contextual fear conditioning task in which mice learn an association between a context and an aversive event (i.e., the delivery of a mild footshock). When returned to the same context, contextual fear memory is inferred from an increase in freezing behavior [15]. The advantage of this task is that a single training episode produces robust memory that is easily-quantifiable and long-lasting [16]. During training wild-type (WT) mice (F1 from a cross between C57B6/N and 129) received 5 footshocks, and then were tested either 1 day or 36 days later (**Figure 2A**). As expected, conditioned freezing levels in trained mice were similar at both the short and long retention delay (planned, unpaired t-test: *t*(14) = 1.31, *P* = 0.21), indicating that no forgetting occurred across this time period. Control groups, that underwent the same procedure but did not receive footshocks during training, showed little freezing during testing (**Figure 2B**) (2 way between subjects ANOVA; main effect of training only, *F*(1,28) = 63.08, *P*<0.0001).

Neuronal stimulation is associated with increases in intracellular calcium levels through NMDA receptor activation or voltage-gated calcium channels. Because these increases in calcium lead to the rapid upregulation of activity-regulated genes such as *c-fos*, measuring Fos protein levels allows for the detection of recently activated neurons [11], [12], [17] (see **Supplementary Note in Text S1**). Therefore, in order to characterize regional activation following contextual fear testing, Fos was quantified in 84 brain regions (**Figure S1**). These included cortical, thalamic, hippocampal, cerebral and midbrain nuclei and were defined according to a standard mouse brain atlas [18] (for complete listing of brain regions, see **Table S1**). In order to isolate changes in gene expression associated with contextual fear memory recall and control for non-specific aspects of the testing procedure, the density of Fos positive cells in each brain region in trained mice was compared to control animals that did not receive footshocks during conditioning. Importantly, multivariate task partial least squares (PLS) analyses revealed that patterns of Fos expression depended both on the training condition as well as the retention delay (significant condition × delay interaction (*P*<0.05). To better we understand the nature of the interaction we conducted separate ‘post-hoc’ analyses. These revealed distinct patterns of Fos expression in the trained vs. control (no shock) mice following testing at both the 1 day (*P*<0.05; **Figure 2C**) and 36 day (*P*<0.01; **Figure 2D**) delay. At the 1 day retention delay, these distinct patterns of activation were primarily driven by increases in Fos expression in the hippocampus (CA1-d, CA1-p and DG-low) in trained mice (**Figure 2C**). In contrast, at the 36 day retention delay, distinct patterns of activation were associated with increases in Fos expression in distributed brain regions in trained mice. These included cortical (PrL, Cg-a), thalamic (Hb, C), hypothalamic (LH, SHy, LPO and MPA) regions, as well as the BST, Cpu-dm, AcbSh, VP, LSI, LSV and PAG (**Figure 2D**). Therefore, these data indicate that memory recall is associated with the induction of Fos, and patterns of Fos expression in trained mice are distinct from those in controls.

### Generation of functional networks

Memory recall is thought to involve coordinated activation of multiple brain regions. Therefore, examination of how activity co-varies across brain regions has been used to study these large scale interactions in both human and experimental animal imaging studies and define functional networks engaged during memory recall. In human imaging studies regional brain activity can be assessed by measuring an electrical signal (EGG, MEG) or indirectly by measuring a metabolic signal such as blood flow (fMRI). Functional connectivity is then typically evaluated by assessing covariance of these signals across different brain regions during performance of a given task. In contrast, in experimental animal studies, neuronal activity is typically assessed post-mortem by evaluating, for example, changes in expression of the activity-regulated genes such as *c-fos*. As such measures provide a single index of activation per region per animal, functional connectivity is necessarily estimated by computing covariance across subjects, rather than within subjects. Both within and across subjects covariance can be used to infer interactions between neural elements [9], [19].

**Figure 3:**
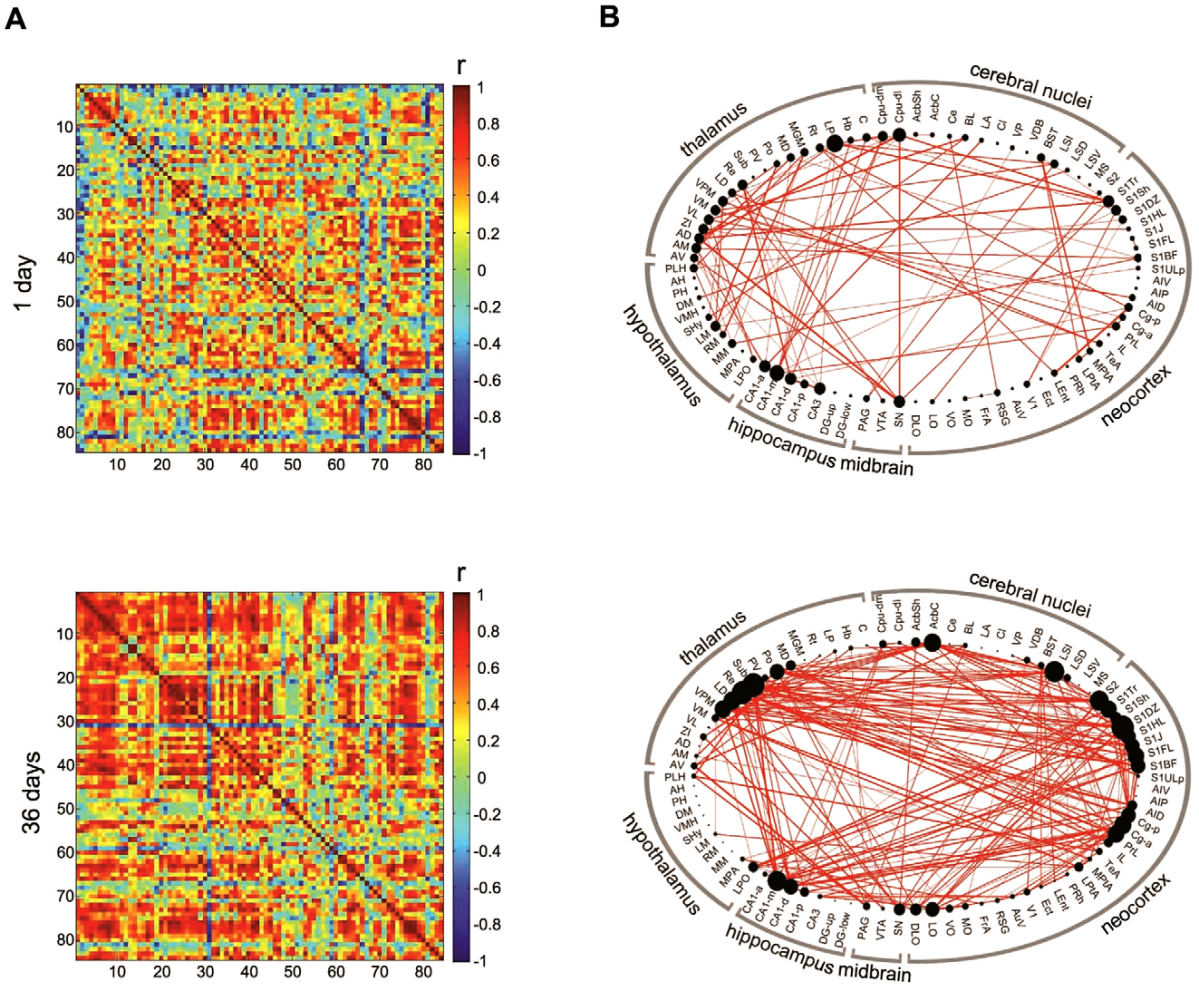
Generation of long-term fear memory networks. **A**. Matrices showing inter-regional correlations for Fos expression at the short (upper) and long (lower) retention delays. Axes are numbered, and correspond to brain regions listed in **Table S1**. Colors reflect correlation strength (scale, right). **B**. Network graphs were generated by considering only the strongest correlations (Pearson's r≥0.83). In these graphs, regions are grouped by major brain subdivision and node size is proportional to the number of connections (degree) while the weight of the connection is proportional to correlation strength.

Accordingly, we next computed a complete set of inter-regional correlations in the groups of mice tested at each retention delay (**Figure 3A**). This allowed us to identify collections of brain regions where Fos expression co-varied across mice, presumably constituting components of a common network engaged by recall of long-term fear memory [19], [20]. Network graphs for each condition were subsequently generated by considering only the strongest correlations (Pearson's r≥0.83, *P*<0.005) (**Figure 3B**). In addition, networks were generated using either more (r≥0.87, *P*≤0.0025) or less (r≥0.79, *P*≤0.025) conservative thresholds (**Figure S2**) or alternate correlation coefficients (Spearman's rank; **Figure S3**) in order to evaluate whether network properties were stable across a range of conditions. In all resulting undirected graphs, nodes (brain regions) are connected by edges representing super-threshold inter-regional correlations.

The structure of these network graphs varied across conditions, but, importantly, these differences were not simply an artifact of group differences in signal strength and/or variance (**Figure S4**). Moreover, a number of other immediate early genes are regulated by neural activity, including *egr-1* [11]. In order to explore the generality of our effects we additionally quantified Egr-1 expression in a subset of brain regions in the WT/36 day group. We found that Fos- and Egr-1-derived patterns of inter-regional correlations were similar (**Figure S5**), consistent with previous studies showing that immediate early genes are typically expressed in the same or largely overlapping neuronal populations [12]. Finally, while functional connections in these networks reflect statistical (rather than physical) relationships between regions, we found that there was excellent correspondence with known neuroanatomy. For example, in the 36 day retention delay network, comparison of functional connections for one brain region (reuniens thalamic nucleus; Re) with published, anatomical data revealed that all functional connections had corresponding anatomical connections in the primary and high confidence networks (**Figure S6** and **Table S2**). While direct anatomical connections are not necessary for two regions to be functionally connected (e.g., they might be co-modulated by a third region), nonetheless this finding is consistent with previous analyses indicating that there is typically good correspondence between functional and underlying structural connections in brain networks [21].

In our matrices, Fos expression in the anterodorsal thalamic nucleus consistently showed negative correlations with other brain regions. In constructing our networks we focused on only positive correlations. However, while relatively few in number, such negative correlations are interesting, and may reflect repression networks. Indeed, future studies using activity-regulated genes that have higher basal levels of expression (e.g., Egr-1) may offer greater sensitivity at detecting negative correlations (as levels can be regulated bi-directionally).

### Networks change with memory age

Regional inactivation or lesions may disrupt expression of a previously-acquired memory. As these retrograde effects are often temporally-graded (with either newer or older memories differentially affected) [15], [22], [23], [24], these data provide evidence that memory organization changes with memory age (or systems consolidation [25]). In the present experiment, we evaluated long-term contextual fear memory at two time-points after training, and this therefore provides an opportunity to track these changes in network organization at a global level. Accordingly, we categorized our 84 regions into major brain subdivisions (e.g., neocortex, hippocampus, midbrain, cerebral nuclei; **Table S1**) and asked whether connection strength between these major subdivisions differed at the 1 day and 36 day time-points (see **Table S3**). We focused in particular, on three *a priori* predictions of systems consolidation models [26], [27]: Memory aging would be expected to be associated with 1) strengthening of connections between different neocortical modules, 2) a gradual disengagement of the hippocampus and 3) an emergent role for prefrontal cortical regions in memory expression.

**Figure 4:**
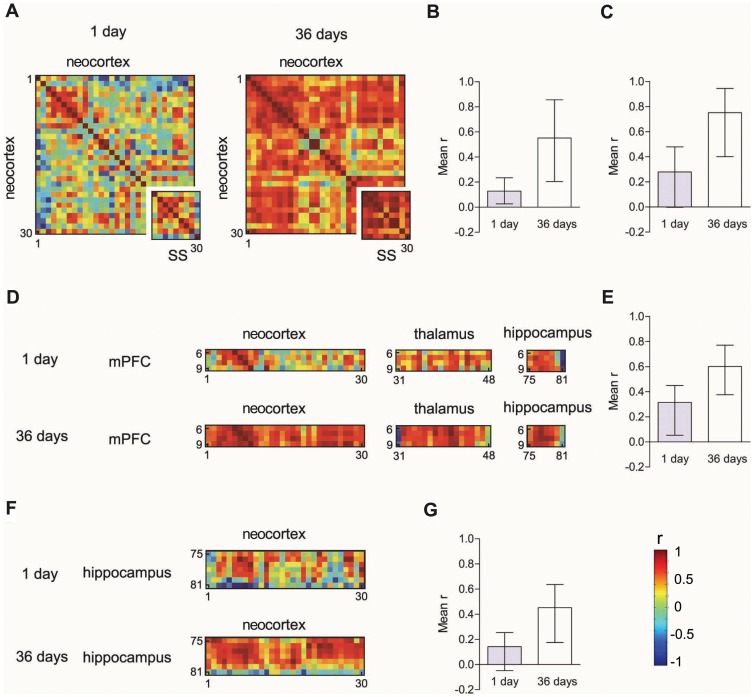
Functional connectivity changes as a function of memory age. **A**. Color-coded matrices showing inter-regional correlations for Fos expression within the neocortex at the short (1 day) and long (36 days) retention delays. The inset matrices correspond to inter-regional correlations for subregions of the somatosensory (SS) cortex. Mean inter-regional correlation coefficients were greater at the long delays for (**B**) cortical regions and (**C**) somatosensory regions (for this, and other comparisons, see **Table S3**). **D**. Color-coded matrices showing inter-regional correlations for Fos expression between regions of the prefrontal cortex and other cortical, thalamic and hippocampal regions at the short (1 day) and long (36 days) retention delays. **E**. Mean correlation coefficients were greater at the long delay, suggesting that medial prefrontal cortex regions play increasingly important roles in memory expression as a function of memory age. **F**. Color-coded matrices showing inter-regional correlations for Fos expression between hippocampal and cortical regions at the short (1 day) and long (36 days) retention delays. **G**. Correlation strength increased over time, suggesting that the hippocampus plays a sustained role in memory expression. In all graphs error bars indicate 95% confidence intervals.

This analysis revealed three main forms of reorganization. First, Fos expression (or activity) among cortical regions was more strongly correlated at the 36-day, compared to 1-day, retention delay (**Figure 4A–B**). This pattern was particularly evident within subdivisions of the somatosensory cortex, where activity was more tightly coupled at the longer retention delay (**Figure 4C**). This increase in inter-cortical correlated activity as a function of memory age is consistent with the idea that consolidation is associated with the strengthening of functional connections between anatomically-distinct regions of the neocortex, leading to the coordination of activity across cortical cell assemblies [6]. Second, previous studies suggested that subregions of the medial prefrontal cortex (including, in particular, the anterior cingulate [Cg-a] [22], [23], [28] and prelimbic cortex [PrL] [23], [29], [30], [31]) play an increasingly important role in memory expression as a function of memory age. Consistent with this, we found an increase in correlated activity between the medial prefrontal cortex and other cortical, thalamic, and hippocampal regions at the longer retention delay (**Figure 4D–E**). Third, whether the hippocampus plays a transient or more sustained role in memory expression is more controversial [26], [27], [32]. While the magnitude of the hippocampal Fos signal declined with retention delay (**Figure 2**), nonetheless we found that this activity either remained tightly coupled or became more tightly coupled with Fos expression in other brain regions following recall at the remote time-point. For example, correlated activity between hippocampal regions and thalamic regions and between hippocampal regions and the midbrain were equivalent at the short and long retention delays (see **Table S3**), whereas coupling of activity between hippocampal and neocortical regions increased over time (**Figure 4F–G**). That hippocampal activity (albeit reduced in magnitude) remains tightly coupled with activity in other thalamic and cortical regions suggests that hippocampal regions play a sustained role in the expression of contextual fear memories. Together, these changes in functional connectivity are consistent with the idea that networks supporting contextual fear evolve as a function of time. These changes in organization are unlikely to be related to changes in behavior since levels of conditioned freezing were similar at both time points. However, as we only measured freezing we cannot exclude that other indices of conditioned fear (e.g., changes in respiration or heart rate) differ at the short and long retention delays.

### Networks engaged by fear memory recall have small-world organization

**Figure 5:**
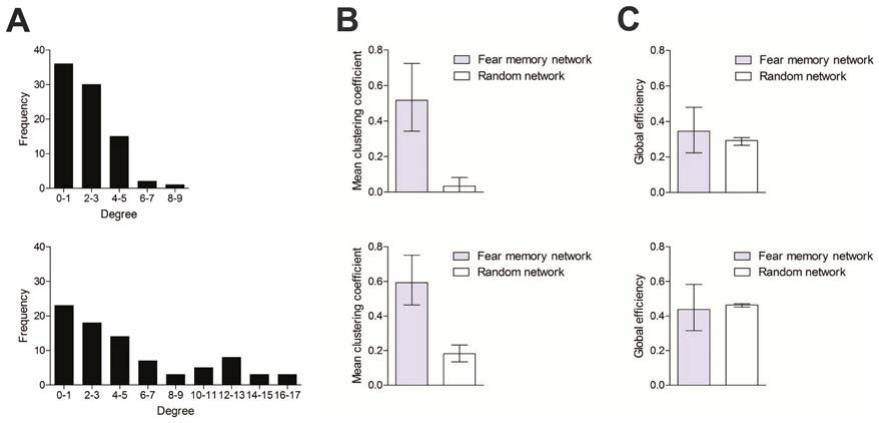
Fear memory networks have small-world structure. **A**. Histogram showing degree distribution for fear memory networks corresponding to the 1 day (upper) and 36 day (lower) retention delay. **B**. Mean clustering coefficient for fear memory vs. random network. At both short (upper) and long (lower) retention delays the fear memory network was more clustered. **C**. Mean global efficiency for fear memory vs. random network. At both short (upper) and long (lower) retention delays global efficiency (or integration) was equivalent in the fear memory vs. random networks. Error bars represent 95% confidence intervals.

Our data suggest that recall of long-term fear memories involves coordinated activation of a broad network of brain regions. Graphical representation of these networks suggest that they are complex in nature, with the majority of brain regions (or nodes) having relatively few connections, but a minority of nodes being highly connected (**Figure 3B**). This non-Gaussian degree distribution was observed at both short and long retention delays (**Figure 5A**), and when either more (r≥0.87, *P*≤0.0025) or less (r≥0.79, *P*≤0.025) conservative thresholds were used to generate the networks (**Figure S7**).

Many complex networks, including both anatomical and functional brain networks, have small-world organization [13], [14]. This type of organization ensures that networks are concurrently segregated—allowing for specialized processing in more densely-connected clusters—and integrated—allowing for efficient information flow through the network [14]. To evaluate whether fear memory networks have similar small-world organization for each network we generated control networks with random topology (matched for node, degree and degree distribution; see **Supplementary Note in Text S1**). To assess segregation we initially computed the clustering coefficient for each network. The clustering coefficient measures the tendency for a node's nearest neighbors to be also connected to each other (quantified as the number of connections between a node's nearest neighbors as a proportion of all possible connections [33]). Using this measure, we found that fear memory networks were consistently more segregated compared to random networks. This was evident at both short and long retention delays (**Figure 5B**) and when more (r≥0.87, P≤0.0025) or less (r≥0.79, P≤0.025) conservative thresholds were used for network generation (**Figure S8**). Moreover, a similar pattern of results was found using alternate segregation measures (local efficiency and transitivity; **Figure S9**).

Integration may be evaluated by quantifying either path length (average shortest path length between all node pairs) or global efficiency (the average inverse path length between all node pairs). Using global efficiency as a measure of integration, we found that fear memory networks exhibited equivalent integration compared to random networks (**Figure 5C**), as would be anticipated for networks with small-world topology. This pattern was evident at both retention delays and across a range of thresholds for network generation (**Figure S10**). Moreover, we found a similar pattern of results using path length as a measure of integration (**Figure S11)**. Therefore, like other macroscale (i.e., inter-regional structural and functional connections) and micro/meso-scale (i.e., intra-regional connectivity) brain networks [14], these analyses indicate that functional networks engaged by recall of long-term fear memories have properties that are consistent with small-world topology.

### Fear memory network includes a densely-connected thalamic-hippocampal- cortical core

**Figure 6:**
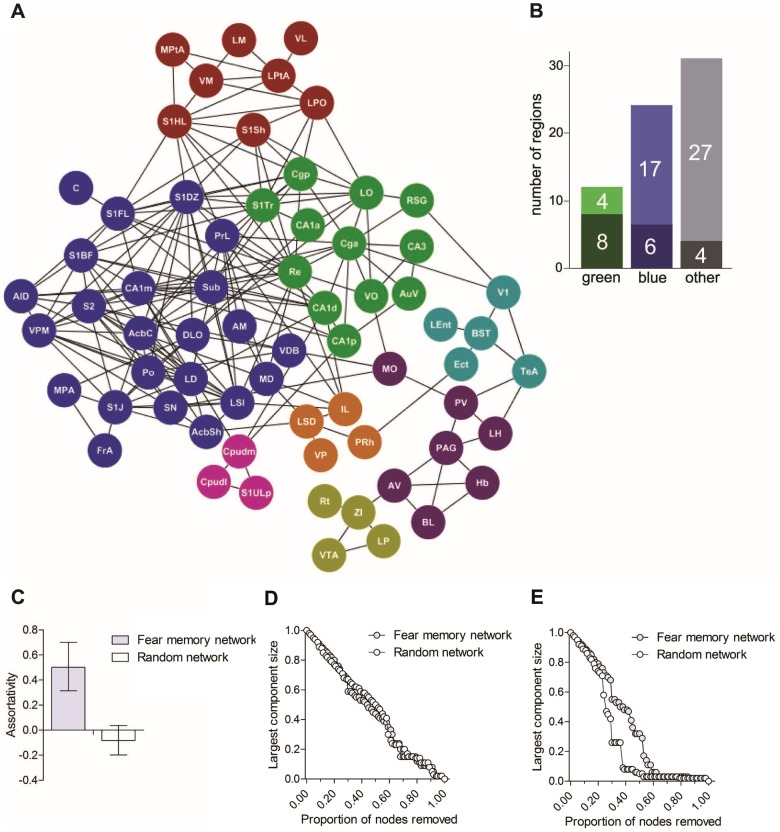
The long-term fear memory network is clustered and resilient. **A**. Network summarizing functional connections after memory recall at the long delay. Brain regions were categorized into discrete (color-coded) clusters with similar connectivity to the rest of the network using the Markov Clustering Algorithm. This network contains a densely-interconnected core (green and blue clusters). **B**. Proportion of brain regions in green, blue and remaining clusters that have previously been implicated in remote memory expression (darkly-shaded portion of bars). Remote memory brain regions are over-represented in the green cluster compared to remaining network (for complete list of regions see **Table S5**). **C**. Assortativity for the fear memory vs. random network. Higher assortativity in the fear memory network indicates that highly-connected nodes tend to be connected to one another. Error bars represent 95% confidence interval. **D**. Consequence of random node deletion on the size of the largest connected component in the fear memory network (grey circles) vs. matched random control network (white circles). In both networks, as nodes were successively removed the size of the largest connected component declined. **E**. Consequence of targeted node deletion on the size of the largest connected component in the fear memory network (grey circles) vs. matched random control network (white circles). In this simulation, nodes were removed in order of descending degree value. The fear memory network was more resilient to successive deletion of high degree nodes. In both graphs, component size is shown as a proportion of the largest original component.

Networks engaged by recall of a long-term fear memory were highly clustered. To examine this structure in more detail we next focused on the network engaged by memory recall at the long retention delay, as this network was densely-connected and fear memory was robustly expressed. We applied the Markov clustering algorithm to systematically organize nodes into discrete modules based on their common inter-connections. This analysis identified eight distinct clusters in the fear memory network, and, in particular, a large densely-connected central component containing two groupings (green and blue nodes; **Figure 6A**). The green grouping consisted almost entirely of hippocampal and cortical regions (**Table S4**) that appear to cluster based on their common interaction with the Cg-a. Interestingly, many of the regions within this grouping, including the Cg-a, have previously been implicated in long-term memory expression, especially at remote time-points following training (**Figure 6B** and **Table S5**). Within the blue cluster, thalamic regions were over-represented (**Table S4**), and, similar to the green cluster, this grouping contained several regions that play important roles in remote memory expression, including PrL, Ca1-m, C, LD and MD (**Figure 6B** and **Table S5**).

Many of these brain regions in the green and blue clusters were highly connected, suggesting that this central core is composed of mutually-interconnected high degree ‘hub’ nodes. We formally evaluated this by computing assortativity—the tendency for nodes with same or similar degree (i.e., the number of connections for a given node) to be directly connected with one another [33], [34]. Compared to control, random topology networks, our fear memory network had a strongly positive assortativity coefficient (**Figure 6C**), consistent with the observation that high degree nodes tend to be connected to one another. Such organization is thought to offer additional resilience, as this central core is more able to withstand removal of multiple hub nodes before network failure. We tested this idea by evaluating network resiliency in our fear memory vs. random network. We simulated the effects of both random node removal and targeted attacks (progressive removal of highest degree nodes) [35], [36]. In both networks, successive random node removal led to gradual disintegration of the network: as nodes were successively removed the size of the largest connected component declined (**Figure 6D**). The rate of disintegration was similar in both networks, suggesting that the fear memory and control networks exhibit equivalent resilience to random node removal. However, the fear memory network was considerably more resilient to targeted attacks. Whereas removal of ∼37% of the most connected nodes led to complete disintegration of the control network, removal of ∼64% of the most connected nodes was necessary to see similar disintegration of the fear memory network (**Figure 6E**).

### Identification of fear memory network hubs

**Figure 7:**
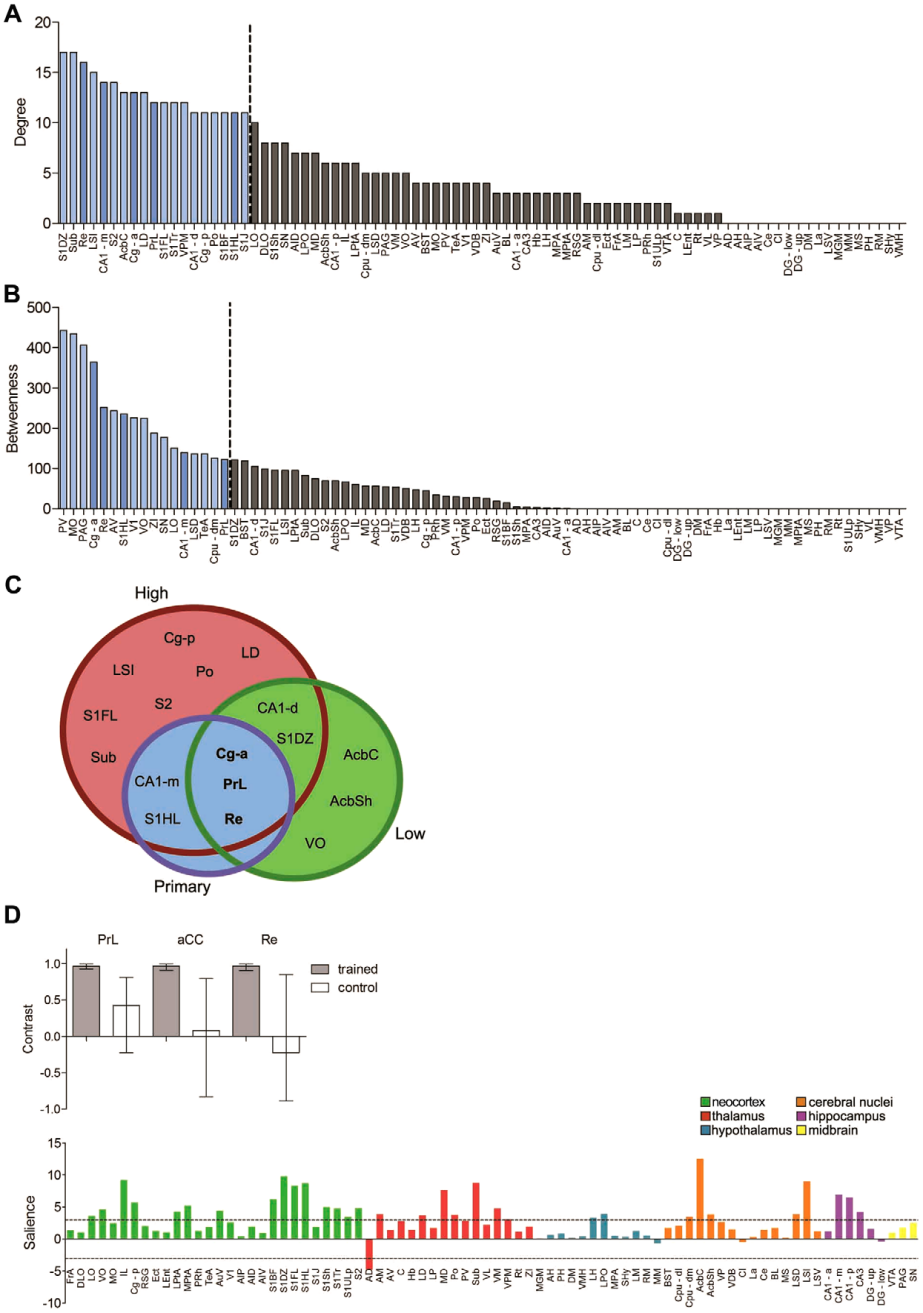
Identification of hub regions in the long-term memory network. Brain regions ranked in descending order for (**A**) degree and (**B**) betweenness. **C**. Venn diagram shows the overlap between brain regions ranked above the 80^th^ percentile for degree and betweenness in each of the primary, high and low confidence networks. **D**. The three putative hub regions (Cg-a, PrL and Re) that were ranked above the 80^th^ percentile for degree and betweenness in all three networks were used as seeds in a multi-seed PLS analysis. These analyses identified a LV (upper graph) that strongly differentiated the patterns of correlations between the seed and other brain regions in the trained (closed bars) vs. control (open bars) conditions. The salience scores (lower graph) identify brain regions whose activity correlates strongly with all three seed regions in the trained (but not control) mice. The hatched lines reflect a salience of ±3, above or below which the contribution of the regions is considered reliable.

Our analyses reveal that expression of a long-term fear memory involves widespread interactions between brain regions. While this is consistent with the idea that these types of memories are distributed, nonetheless highly-connected regions (or hubs) may disproportionately influence network function. In order to identify specific hub regions within our fear memory network, we therefore next ranked all nodes by degree. As expected, nodes within the large, central component were highly ranked. They accounted for the vast majority of regions >80^th^ percentile rank in our primary network (**Figure 7A**), as well as in networks generated using either more (r≥0.87, *P*≤0.0025) or less (r≥0.79, *P*≤0.025) conservative thresholds (**Figure S12**). However, how any given node interacts with the rest of the network depends not only on the number of connections but also the nature of these connections. We therefore additionally ranked nodes using another measure of centrality, betweenness (**Figure 7B** **, Figure S12**). Betweenness centrality computes the number of shortest paths between node pairs that pass through a given node, and nodes that have a large number of intermodular connections tend to have high betweenness centrality [37]. Nodes ranked high in betweenness differ from those that were computed in random networks (**Figure S13**). Of the highly-connected nodes, three regions—Cg-a, PrL, and Re—were also ranked above the 80^th^ percentile in terms of betweenness in each of the low, moderate and high confidence networks (**Figure 7C**), suggesting that this subset of high-degree regions may function as connector hubs and facilitate global intermodular integration.

We next used seed PLS analysis to examine patterns of interactions of the three putative hubs (Cg-a, PrL, and Re) (**Figure 7D**). These analyses verified that these regions interacted most strongly with cortical, thalamic, and hippocampal regions, consistent with the network graphs. Most importantly, this pattern of interactions differentiated the fear memory and control conditions (*P*<0.001), suggesting that regions that show reliable connectivity with the seed regions form a functional network that is engaged during contextual fear memory expression specifically, and not engaged by other non-specific factors of the testing procedure.

### Altered fear memory networks in α-CaMKII^+/−^ mice with accelerated forgetting

**Figure 8:**
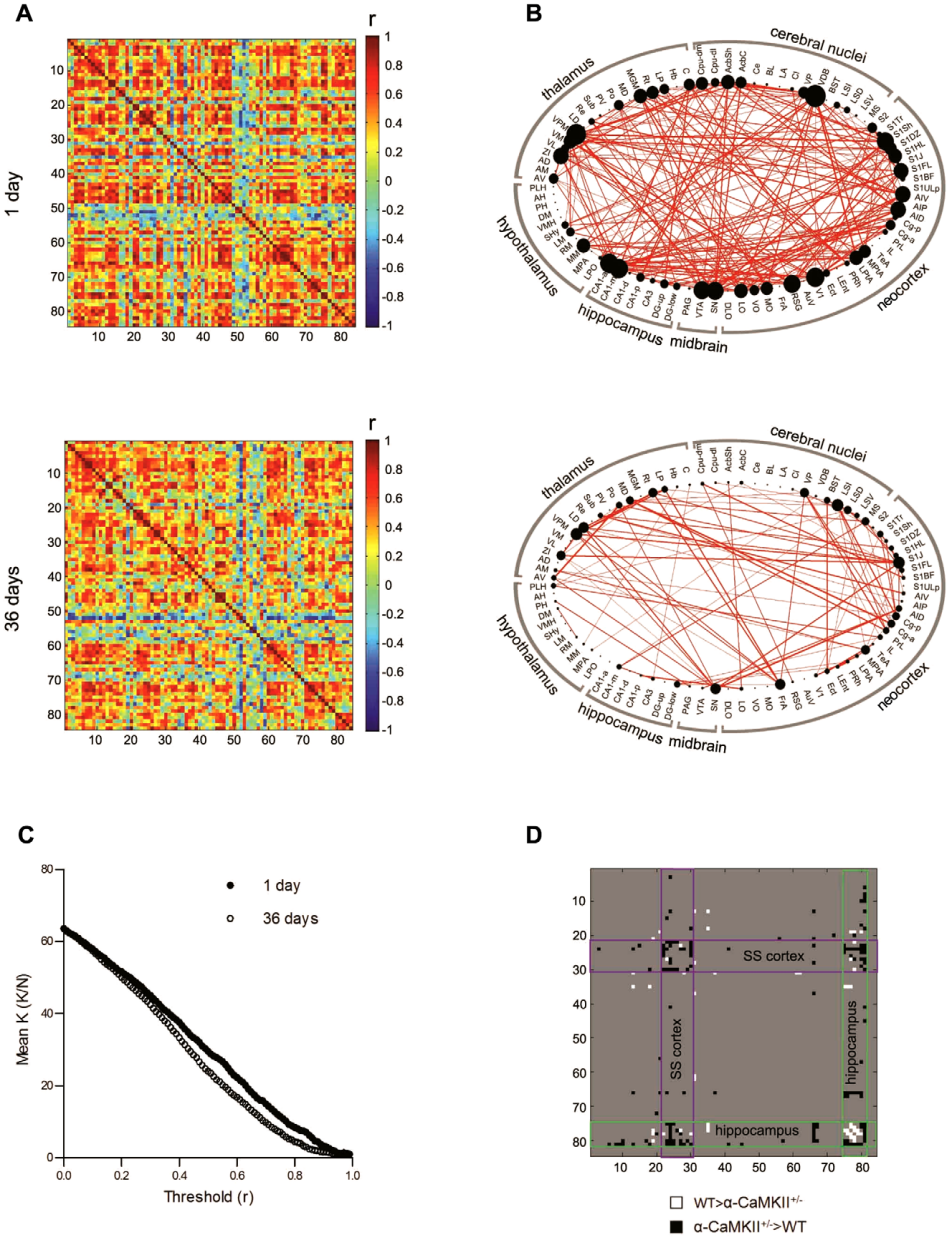
Fear memory networks are altered in α-CaMKII^+/−^ mice. **A**. Matrices showing inter-regional correlations for Fos expression at the 1 day (upper) and 36 day (lower) retention delays for α-CaMKII^+/−^ mice. Axes are numbered, and correspond to brain regions listed in **Table S1**. Colors reflect correlation strength (scale, right). **B**. Network graphs generated for Pearson's r≥0.83. In these graphs, regions are grouped by major brain subdivision and node size is proportional to the number of connections (degree) while the weight of the connection is proportional to correlation strength. **C**. Comparison of mean degree (observed connections per node [K]/all possible connections for that node [N]) for α-CaMKII^+/−^ mice at the short (1 day; closed circles) and long (36 days; open circles) retention delays. The number of connections per node is greater at the 1 day delay for almost all correlation (r) thresholds, reflecting greater network density at the shorter delay. **D**. Individual correlations differ between WT and α-CaMKII^+/−^ matrices. These were determined by permutation testing, using a false discovery rate of 5% to account for multiple comparisons. Correlations where WT>α-CaMKII^+/−^ are shown in white, and where α-CaMKII^+/−^>WT are shown in black. Notably, at this short retention delay correlation strength within the somatosensory cortex (outlined in purple) and between hippocampal regions and other brain regions (outlined in green) was stronger in α-CaMKII^+/−^ mice.

Usually, fear memories are very stable, and can be maintained over extended periods with little decay [16]. However, α-CaMKII^+/−^ mice exhibit accelerated forgetting [22], and therefore provide an opportunity to contrast initial organization of functional networks for fear memories that are destined to persist with those that are destined to fade with time. Accordingly, we trained α-CaMKII^+/−^ mice (in parallel with their WT littermate controls, described above) and tested their memory 1 or 36 days later. As expected, the mutants exhibited accelerated forgetting, with freezing levels greatly reduced at the longer (12.4±5.2%) compared to shorter (49.5±10.5%) retention delay. Importantly, freezing levels at the shorter retention delay were similar to WT mice (66.5±8.7%; planned, unpaired t-test, *t*(14) = 1.25, *P* = 0.23). An ANOVA confirmed the differential impact of this mutation on memory at the longer retention delay (2 way between subjects ANOVA; delay × genotype interaction, *F*(1,28) = 10.62, *P*<0.001). Following testing, Fos expression was subsequently quantified in 84 brain regions, and networks generated as previously described (**Figure 8A–B**).

Similar to WT mice, fear memory networks in the α-CaMKII^+/−^ mice had a non-Gaussian degree distribution and small-world topology. As in the WT networks, the majority of brain regions had relatively few connections, but a minority of nodes was highly connected (**Figure S14A**). Moreover, compared to random control networks, mutant networks were more segregated, but equivalently integrated, at both the short and long retention delays (**Figure S14B–C**). Finally, network density declined (rather than increased) as a function of time most likely reflecting no (or very weak) memory expression in the mutants at the remote retention delay (**Figure 8C**).

While general network organization was similar in WT and α-CaMKII^+/−^ mice, nevertheless there were some notable differences. In particular, at the 1-d retention delay, while WT and α-CaMKII^+/−^ mice exhibited equivalent levels of conditioned fear, functional connections differed within the neocortex (and especially the somatosensory cortex), within the hippocampus and between the hippocampus and neocortex between groups (**Figure 8D**). These differences between WT and α-CaMKII^+/−^ networks at this shorter retention delay indicate equivalent behavior may be generated by distinct networks. As the α-CaMKII^+/−^ mice showed accelerated forgetting, they further suggest that such degenerate solutions may not always be so robust. Indeed, they raise the possibility that functional connections within the neocortex, within the hippocampus and between the hippocampus and neocortex are especially important for memory stability.

## Discussion

Here we used global mapping and graph theoretical approaches to characterize functional networks engaged by recall of a long-term memory in mice. These experiments yielded four principal findings. First, like many real-world networks, functional networks engaged by recall of long-term memory have a small-world topology. This type of organization ensures that networks are concurrently segregated—allowing for specialized processing in more densely-connected clusters—and integrated—allowing for efficient information flow through the network [14]. Second, while networks maintain small-world topology across time, the specific nature of functional connections changes as a function of memory age. Memory aging was associated with strengthening of inter-cortical functional connections and with strengthening of connectivity between prefrontal areas and thalamic, cortical and hippocampal areas. Third, the functional network supporting long-term fear memory had a characteristic thalamic-hippocampal-cortical signature. Many thalamic, hippocampal and cortical regions formed a central core of mutually-interconnected regions, and the dense interconnectivity of these high-degree regions likely enhances network resilience. Fourth, several regions (including the anterior cingulate cortex and prelimbic cortex) were identified as important hubs within this long-term memory network and therefore might play disproportionately important roles in network function and resilience. While previous imaging and electrophysiological studies have examined the role of specific regions and small collections of regions in expression of a long-term memory, this analysis provides global picture of how a long-term fear memory is organized in mice.

In our experiments, we evaluated contextual fear memory at both short and long delays after training. We found that functional networks differed at these two time-points, consistent with the idea that memory organization changes as a function of memory age, a process known as systems consolidation [25]. One prominent model of systems consolidation [27] proposes that memories are initially encoded in hippocampal-cortical networks, and that reactivation of these networks then leads to the incremental strengthening of inter-cortical connectivity, and an emergent role for prefrontal cortical regions in memory expression coupled with disengagement of the hippocampus. Consistent with this model, we found that functional connections within the neocortex strengthened over time, and this was especially evident within the somatosensory cortex. Moreover, functional connections between prefrontal cortical regions (including Cg-a and PrL) and thalamic, posterior cortical and hippocampal regions were stronger at the longer retention delay, supporting the idea that these regions play important roles in the memory expression at remote time points after encoding [22], [23], [29], [38], [39]. However, the picture in the hippocampus was more complex. At the remote time-point, hippocampal activity was reduced in magnitude, yet remained tightly coupled with activity in other thalamic and cortical regions. Clearly, consideration of Fos expression levels alone may have suggested that the hippocampus is not important at remote time points. However, the co-activity data paint a more nuanced picture of the role of the hippocampus in expression of remote contextual fear memory: That hippocampal activity (albeit reduced in magnitude) remains tightly coupled with activity in other thalamic and cortical regions suggests that hippocampal regions play a sustained role in the expression of contextual fear memories. This more sustained role is consistent with the finding that light-induced inactivation of CA1 neurons blocks expression of remote contextual fear memories [39], as well as conceptualizations that the hippocampus is re-engaged regardless of the age of the context memory as proposed in alternate models [26] (see also: [32]).

What might these changes in network organization reflect? Levels of freezing were similar at the short and long delays, and therefore distinct networks are unlikely to be directly related to different levels of fear. However, while behavioral output may be similar at these two time points, it is possible that underlying context representations differ. Such representational changes are suggested by time-dependent increases in context generalization following fear conditioning [40]. Indeed, using an identical training protocol, we found that mice froze more when tested in the training vs. a novel context at the short delay, but froze equivalently when tested in these two contexts at the longer delay (see **Figure S15**). Therefore, time-dependent changes in the nature of functional connections might reflect the transformation of the contextual fear memory from a precise, detailed form into a less precise, generalized form [40]. A second possibility is that network changes reflect an interaction between new and existing memories. In addition to changes in the nature of connections, the density of connections also increased as a function of retention delay. This increase in connection density suggests that recall of an older fear memory involves the coordinated activation of a broader network, perhaps reflecting the engagement of a more expansive associative net. Such time-dependent network expansion is predicted by models that propose that consolidation involves, in part, the integration of new information into existing knowledge bases or schemas [41], [42].

Fear memory networks had a distinct thalamic-hippocampal-cortical signature, and this was most pronounced at the longer retention delay. Interestingly, electrophysiological recording studies in awake, behaving animals suggest similar networks are engaged during encoding and subsequently re-engaged during post-encoding periods such as sleep or even retrieval [43]. For example, encoding-related situations (e.g., spatial exploration or exploration-based learning) are associated with coordinated activation of the hippocampus and neocortex, with especially pronounced coupling between the hippocampal and prefrontal cortical activity [44], [45], [46]. Moreover, these same patterns of hippocampal and cortical activity are spontaneously replayed in subsequent ‘offline’ periods such as quiet wakefulness or sleep [47], and hippocampal-cortical activity remains coupled [10], [48]. Moreover, similar coupling of hippocampal and prefrontal activity is observed during retrieval of a contextual fear conditioning [49]. While hippocampal-cortical replay has been studied in most detail, post-encoding replay also been observed hippocampal-thalamic and hippocampal-striatal circuits [50], [51], suggesting that replay occurs in brain-wide networks. This brain-wide reactivation is thought to play an essential role in systems consolidation, leading to the incremental remodeling of networks for long-term memory storage. Indeed, disruption of replay impairs consolidation of spatial memory [52], [53].

In our networks, functional connections reflect statistical, rather than necessarily physical, relationships between brain regions. Accordingly, our network analyses allowed us to generate hypotheses about which brain regions may play privileged roles in memory expression, with highly-connected hub-like regions likelier to influence overall network function. In particular, our analysis revealed that the network engaged by recall of a fear memory more than one month after training contains a densely-interconnected central core of highly-connected hub-like regions. Importantly, this central core included many brain regions that have previously been implicated in the expression of contextual fear memories at such remote time-points [25] (**Table S4**), consistent with the idea that hubs play more integral roles in network function. For example, loss of function manipulations (including pharmacological inactivation, cytotoxic lesions or optogenetic silencing) targeting the anterior cingulate cortex [22], [28], [39], [54], hippocampus (including CA1 and CA3 fields) [39], [55], retrosplenial cortex [56] and prelimbic cortex [30] disrupt expression of contextual fear memories at extended retention delays. Similarly, loss of function manipulations targeting these regions, as well as thalamic (e.g., LD, MD, C) and orbitofrontal (DLO, VO, LO) regions affect expression of other forms of remote memory, including spatial and olfactory-based memories [23], [57], [58]. While correspondence between the predictions derived from the network analysis and these published findings provides some validation of our approach, perhaps greater value lies in using this approach for the identification of novel, candidate brain regions. In this regard, it is interesting that many thalamic regions were identified within this central core, including Re, Sub, VPM and Po. These regions, by virtue of their widespread connectivity with both the hippocampus and neocortex, are ideally positioned to influence network function. In particular, thalamic regions strongly influence cortical activity by modulating up/down states [59] and therefore may play central roles in coordinating memory replay and retrieval. Indeed a recent lesion study provided evidence that Re plays a key role in remote memory [60].

One interesting observation was that the same (or very similar) behavioral output was associated with distinct functional networks, suggesting that there is a degree of degeneracy at the network level (i.e., an ability of distinct constellations of neural elements to produce same output [61], [62]). This was the case for networks engaged by recall of contextual fear memory at the short vs. long delay in WT mice. Likewise, α-CaMKII^+/−^ mice exhibited equivalent levels of conditioned freezing compared to WT littermates at the short delay, yet underlying functional networks differed considerably. Together, this suggests that a population of network solutions may support contextual fear memory, and raise two related issues. First, because of this degeneracy, pre-training manipulations of key brain regions may not necessarily prevent the formation of a contextual fear memory. For example, pre-training lesions of the hippocampus surprisingly do not always impair acquisition of a contextual fear memory [63], [64], and this is presumably because alternate networks can support this type of memory in the absence of an intact hippocampus at the time of training. If multiple networks can support the formation of a contextual fear memory, then a second issue is whether all networks perform equivalently under all conditions? In the case of animals trained without a hippocampus this does not seem to be the case. While hippocampal lesioned animals may form a contextual fear memory, acquisition is less efficient [64], and the resultant memory is less precise [63] and less durable [65]. Similarly, in our study, the α-CaMKII^+/−^ mice were able to form a contextual fear memory, but this memory faded rapidly over time. This highlights that not all degenerate solutions necessarily have the same robustness, although it remains to be determined which specific alterations in network organization in α-CaMKII^+/−^ mice might be causally related to premature forgetting.

In these studies we quantified expression of the immediate early gene, *c-fos*, to track changes in neural activity. Like other IEGs, Fos is induced rapidly in neurons after sustained activity and can be quantified at single-cell resolution across the entire brain [12], [66]. However, as Fos is an indirect measure of activity, there some potential limitations worth noting. Foremost, promoter-specific differences in regional and cellular expression mean that it is unlikely that there is a uniform relationship between neural activity and Fos across the brain, and regional differences in magnitude of the behaviorally-induced Fos signal inevitably would compromise mapping and subsequent network generation. However, regional differences in signal did not appear to be limiting as correlation strength (or connectivity) was not strongly influenced by Fos signal. A closely-related issue is whether networks based on different activity markers might differ markedly from one another. Accordingly, in additional analyses we quantified expression of another activity-regulated gene, *egr-1*, in a subset of brain regions. While there are inevitably some regional differences in Egr-1 and Fos expression following memory recall, we found that the overall patterns of inter-regional correlations derived from Egr-1 vs. Fos expression were very similar, consistent with idea that many activity-regulated genes are expressed in the same subpopulations of neurons after regional activation [12]. Future refinement of this approach might focus on improving temporal resolution (e.g., examining mRNA for single or multiple activity markers) [11] and data acquisition and analysis (e.g., optical projection tomography or 3D digital reconstruction from serial block-face scanning of activity marker expression [67], [68], [69] and derivation of voxel-based, rather than region-based maps of co-activity). Indeed, a limitation of the current study was the relatively small sample sizes (∼8 mice/group). In the future, implementation of these more high-throughput data acquisition and analysis methods, in particular, should make it is feasible to use larger sample sizes and consequently maximize the power of the approach.

## Methods

### Ethics statement

All experimental protocols were approved by the Animal Care Committee at The Hospital for Sick Children.

### Behavioral and immunohistochemical procedures

In these experiments, Fos and Egr-1 expression was analyzed following recall of long-term contextual fear memory at two time-points after training. A sub-analysis of these brain sections was previously published in which gene expression was quantified in six brain regions [22]. In the current analyses, quantification was extended to 84 brain regions, and the network properties of fear memory were analyzed using graph theoretical approaches. The genetic background of all mice used in these experiments was 50% C57Bl/6NTacfBr and 50% 129Sv/J. Briefly, experimental mice were generated by crossing α-CaMKII^+/−^ mutants that were 50% C57Bl/6NTacfBr and 50% 129Sv/J with mice that were F1 hybrids of the same genetic background. Male and female littermate WT and α-CaMKII^+/−^ mice were used in experiments. All mice were maintained on a 12 h light/dark cycle with free access to food and water. Behavioral experiments were conducted during the light phase of the cycle, and mice were at least 8 weeks old at the time of training.

Context fear conditioning experiments were conducted in a windowless room containing four conditioning chambers. The chambers were stainless steel (31 cm×24 cm×21 cm; Med Associates, St. Albans, VT). The shock-grid floor consisted of stainless steel shock-grid bars (diameter 3.2 mm) that were spaced 7.9 mm apart. The front, top, and back of the chamber were made of clear acrylic and the two sides made of modular aluminum. Mouse freezing behavior was monitored via four overhead cameras. Freezing was assessed using an automated scoring system (Actimetrics, Wilmette, IL), which digitized the video signal at 4 Hz and compared frame by frame movement to determine the amount of time spent freezing. During training, WT and α-CaMKII^+/−^mice were placed in the conditioning chamber for seven minutes. After two minutes they were presented with five unsignalled footshocks (2 s duration, 0.75 mA, 1 minute apart). Following the last footshock mice remained in the context for an additional minute, and then were returned to their home cage. Separate groups of mice were tested either 1 day (WT mice, n = 8, CaMKII^+/−^ mice, n = 8) or 36 days (WT mice, n = 8, CaMKII^+/−^ mice, n = 8) later. During testing, mice were placed back in the conditioning chamber for two minutes, and freezing was assessed. Control mice underwent the same procedure but did not receive the footshocks during the conditioning session (WT mice, 1 day delay, n = 8, WT mice, 36 day delay, n = 9).

Ninety minutes following the completion of testing, mice were deeply anesthetized then perfused transcardially with phosphate buffered saline (PBS) followed by 4% paraformaldehyde (PFA). The brains were removed, fixed overnight in PFA, then transferred to 30% sucrose solution and stored at 4°C. Fifty µm coronal cryostat sections were cut from the anterior to the posterior of the brain. Every fourth section contributed to a set, creating four sets with sections spaced 200 µm apart. One set was used for Fos and another for Egr-1 immunohistochemical staining. Brains were prepared for immunocytochemistry using an anti Fos (1∶20000) or an anti Egr-1 (1∶7500) primary rabbit polyclonal antibody. A biotinylated goat anti-rabbit antibody (1∶2000) was used as a secondary antibody. Staining was revealed using the avidin-biotin peroxidase method (ABC kit) coupled to diaminobenzidine as a chromogen, as previously described [22].

### Immediate early gene quantification

Fos expression was analyzed in 84 brain regions (see **Table S1**) and included cortical, thalamic, hippocampal, cerebral and midbrain nuclei. The borders of regions were defined manually according to the Franklin and Paxinos mouse brain atlas [18]. This was accomplished at low magnification (2× objective), using the trace contour function in the Stereo Investigator imaging system (Microbrightfield Inc., Colchester, VT). Images were subsequently acquired using either a Nikon Eclipse 80i microscope (Nikon, Instruments, Melville, NY) or Olympus BX61 epifluorescent microscope (Olympus America, Center Valley, PA) at a higher magnification (10× objective) for offline quantification of Fos-positive or Egr-1-positive cells (**Figure S1**).

Quantitative analysis of Fos-positive and Egr-1-positive nuclei was performed on 8-bit grey scale images using Image J software (National Institute of Health, Bethesda, MD). The threshold for detection of positive nuclei was set at a consistent level for each brain region, and only nuclei 25–125 µm^2^ in area were counted. Immunoreactive nuclei were counted in all 84 regions of interest by an experimenter blind to the condition and expressed as the average number of Fos or Egr-1 positive cells per standard unit of area (10,000 µm^2^). For the majority of brain regions three sections were quantified bilaterally and then a mean count was computed for each animal. These sections were evenly spaced, and typically did not include the most anterior and posterior portions of a region in order to avoid sampling outside the region of interest. In a small subset of regions, counts were based on <3 sections, usually because the anterior-posterior extent of the region ≤600 µm (e.g., VTA).

### Task PLS analysis

PLS is a multivariate statistical technique that is used to identify optimal patterns of functional activity or connectivity that differentiate conditions [9]. Task PLS is used in the analysis of brain region activity to describe the relationship between experimental conditions and functional activity [70]. PLS is able to pull out similarities and differences between groups by identifying brain regions where activation varies with the experimental condition. Through singular value decomposition, PLS produces a set of mutually orthogonal latent variable (LV) pairs. One element of the LV depicts the contrast, which reflects a commonality or difference between conditions. The other element of the LV, the brain region salience, identifies brain regions that show the activation profile across tasks, indicating which brain areas are maximally expressed in a particular LV.

Statistical assessment of PLS was performed by using permutation testing for LVs and bootstrap estimation of standard error for the brain region saliences. For the LV, significance was assessed by permutation testing; resampling without replacement by shuffling which condition (trained vs. control) each mouse was assigned to. Following each resampling, the PLS was recalculated. This was done 500 times in order to determine whether the effects represented in a given LV were significantly different to random noise. For brain region salience, reliability was assessed using bootstrap estimation of standard error. Bootstrap tests were performed by resampling 500 times with replacement, while keeping the subjects assigned to their conditions. This reflects the reliability of the contribution of that brain region to the LV. Brain regions with a bootstrap ratio greater than 3 (roughly corresponding to a confidence interval of 0.01) were considered as reliably contributing to the pattern. An advantage to using this approach over univariate methods is that no corrections for multiple comparisons are necessary because the brain region saliences are calculated on all of the brain regions in a single mathematical step [70].

### Functional connectivity analysis

Within each of the four experimental groups of animals (WT/1 day, WT/36 days, α-CaMKII^+/−^/1 day, α-CaMKII^+/−^/36 days), all possible pairwise correlations between the Fos signal in the 84 regions were determined by computing Pearson correlation coefficients (totalling 3486 correlations). Each complete set of correlations was computed from a vector of size 8, and were displayed as color-coded correlation matrices using Matlab software (Mathworks, Natick, MA). A correlation matrix was also constructed for analysis of Egr-1 expression in 8 regions in the WT/36 day group.

### Wild-type correlation matrix comparisons

In order to evaluate how functional connectivity changed as a function of memory age in WT mice, we categorized our 84 brain regions into major brain subdivisions (hippocampus, thalamus, hypothalamus, cerebral nuclei and neocortex). We then contrasted mean correlation strength between different major subdivisions at the short vs. long retention delays. These contrasts were selected *a priori* according to models of systems consolidation [27] that predict time-dependent changes in the strength of hippocampal, prefrontal cortical and inter-cortical functional connections. Specifically, to assess time-dependent changes in hippocampal functional connectivity, mean correlations were calculated between hippocampal subregions (CA1-a, CA1-m, CA1-d, CA1-p, CA3, DG-up, DG-low) and the other major subdivisions of the brain (thalamus, hypothalamus, cerebral nuclei, midbrain and neocortex). To assess changes in medial prefrontal cortical functional connectivity, mean correlations were calculated between regions in the medial prefrontal cortex (Cg-a, Cg-p, IL, PrL) and the other subdivisions of the brain (thalamus, hypothalamus, cerebral nuclei, midbrain and neocortex). To assess changes in inter-cortical connectivity mean correlations were calculated within the entire neocortex, as well as within only the somatosensory cortex. Ninety-five % confidence intervals were computed by bootstrapping which involves resampling subjects with replacement 1000 times and each time recalculating the mean correlation. Differences between mean correlation coefficients at the 1 and 36 day delay were assessed by calculating the 95% confidence interval of the difference between mean correlations, and correlation differences where this confidence interval ≥0 were considered reliably different.

### Functional network construction

Networks were constructed by thresholding inter-regional correlations in each group of animals. The primary networks were constructed by considering correlations with Pearson's r ≥0.8343, which corresponds to a one tailed significance level of *P*<0.005, uncorrected for multiple comparisons. Higher and lower confidence networks were constructed as well to insure that network properties were not dependent on threshold level selection. A threshold of r≥0.8697 (corresponding to a significance level of *P*<0.0025 [one tailed, uncorrected]) was used to generate the high confidence networks and a threshold of r ≥0.7887 (corresponding to a significance level of *P*<0.01 [one tailed, uncorrected]) was used to generate the low confidence networks. In addition, networks were constructed using Spearman's rank correlation coefficient, with a threshold of r ≥0.8343, which corresponds to a one tailed significance level of *P*<0.005. The nodes in the networks represent brain regions and the correlations that survived thresholding were considered connections. NetDraw (Analytic Technologies, Lexington, KY) was used to visualize networks, with node size set proportional to degree (number of connections) and connection line weights reflecting the strength of the correlation. While potentially interesting, we did not consider negative correlations in the current network analyses.

### Graph theory analysis

Graph theoretical measures were used to characterize properties of the long-term memory networks. For each primary, low and high confidence fear memory network, 1000 random, null hypothesis networks were generated with the same number of active nodes, connections and same degree distribution. Then long-term memory network properties were contrasted with averaged values from these corresponding random, control networks. Network measures and random null hypothesis networks were generated using functions from Olaf Sporns' brain connectivity toolbox (https://www.brain-connectivity-toolbox.net/). Definitions and formulae for these graph theory measures have been described in elsewhere [33]. Ninety-five percent confidence intervals for the network measures are reported in order to determine whether network properties differ reliably between fear memory network and random, control networks. Means and confidence intervals for the network measures were derived by bootstrapping which involves resampling subjects with replacement one thousand times and recalculating the network measures.

#### Integration

Integration in a brain network gives rise to coordinated activation of distributed neuronal populations and brain areas [33]. In our analyses we computed two measures of integration: characteristic path length and global efficiency. Shortest path length is the mean number of connections that need to be traversed to get from one node to another. The characteristic path length is then the average shortest path length for all pairs of nodes in the network [13]. Global efficiency is the average inverse shortest path length in the network [71]. Global efficiency differs from characteristic path length in that it considers unconnected node pairs in its calculation since the inverse of infinity is zero.

#### Segregation

Segregation in a brain network allows for specialized processing in more densely-connected clusters [33]. In our analyses we computed three common measures of segregation: mean clustering coefficient, transitivity and local efficiency. The clustering coefficient is computed by dividing the number of existing connections among a node's directly connected neighbours by the number of possible connections between them [13]. The mean clustering coefficient for the network is then the average clustering coefficient for all of the active nodes in the network. Transitivity is a weighted version of the clustering coefficient that is less biased by low degree nodes [72]. The local efficiency is the global efficiency computed among all directly connected neighbours of a node [71], and the mean local efficiency of all active nodes in the network was considered.

#### Identification and characterization of clusters

Cluster analysis was conducted with the Markov Cluster Algorithm (inflation parameter set at 2.6), a scalable, unsupervised cluster algorithm for networks based on simulation of stochastic flow in graphs (http://www.micans.org/mcl/). Additionally, we validated our results using a number of alternate clustering algorithms, including community clustering, clusterONE, affinity propagation, connected components and k means clustering. These yielded mostly overlapping results. However, we chose to present results based on Markov clustering since previous studies indicated that this procedure is significantly more tolerant to noise and behaves more robustly than other algorithms [73]. Clusters were visualized using Cytoscape (http://www.cytoscape.org/). The assortativity coefficient is a correlation coefficient between the degrees of all directly-connected node pairs. A positive assortativity coefficient indicates that nodes tend to link to other nodes with the same or similar degree [34].

#### Hub identification

Hub regions play disproportionately important roles in the function of a network [33]. Two measures of centrality, degree and betweenness, were computed for all nodes in the network and used to identify candidate hub regions. Degree corresponds to the number of edges that are incident upon a node. Node betweenness is the number of all shortest paths in the network that pass through a given node, and nodes with high values of betweenness centrality participate in a large number of shortest paths. All regions were ranked by degree and betweenness, and candidate hub regions were considered to be regions that were ranked >80^th^ percentile for both measures in primary, as well as high and low confidence, networks. For comparison betweeness values were also computed in 100 random networks.

#### Resilience of networks to sequential node attack

We assessed network resilience to random error and targeted attack using an approach previously described by Achard et al (2006) [36]. Random error was modeled by sequentially removing nodes and all of their connections from the network at random and then recalculating the size of the largest connected component (i.e., collection of connected nodes) in the new network. Targeted network attack was simulated using the same process except that node removal began with the most highly-connected node (i.e., node with the highest degree) and progressed in order of descending degree value. Curves describing the effect of random and targeted node removal on largest component size in the fear memory network were compared to curves for a control network matched for node, degree and degree distribution.

### Seed Partial Least Squares

Seed PLS is used in the analysis of functional connectivity to explore if seed regions exhibit task-related changes in activity in relation to the rest of the brain. Like task PLS described above, seed PLS identifies LVs through singular value decomposition of correlation maps [74]. The LVs maximally describe patterns of brain region interactivity with seed regions that are similar or different between groups. The three brain regions identified as hubs with graph theory measures were used as seeds in this analysis. This analysis was used to verify that activity in hubs co-varies with activity in other brain regions, and to determine if this pattern of interactivity is unique to the experimental condition or shared between the experimental and control condition.

### α-CaMKII^+/−^ and WT 1 day correlation matrix comparisons

In order to compare correlation matrices for the WT and α-CaMKII^+/−^ 1 day retention delay groups, all individual correlations were compared with no *a priori* predictions (3486 comparisons). Significance differences in individual correlations were determined with permutation testing. Permutation testing involved shuffling subject labels to produce several permuted combinations of the original two groups of data. Following each permutation the test statistic (correlation) was recalculated. The number of events that exceeded the observed correlation statistic was determined and a probability of the number of observed events being greater than expected was assigned. The false discovery rate was controlled at the 5% level because of the number of multiple comparisons.

## Supporting Information

Figure S1 **Fos quantification.** Sample images of Fos immunohistochemical staining from a representative brain section. Images were captured with the 2× objective (red border) and 10× objective (blue border). Higher magnification images are shown for specific hippocampal (CA3), cortical (S1Tr), cerebral nuclei (Ce), hypothalamic (DM) and thalamic (VPM) regions. Dotted lines indicate region borders.
(TIF)

Figure S2 **High and low confidence networks in WT mice.** Networks formed from thresholding inter-regional Fos correlations in mice tested at the 1 (upper) and 36 day (lower) retention delay using either a high confidence threshold of r >0.87 (left) or a low confidence threshold of r >0.79 (right). Brain regions are grouped by major brain subdivision and node size is proportional to the number of connections (degree) while the weight of the connection is proportional to correlation strength.
(TIF)

Figure S3 **Spearman rank correlation networks in WT mice.** **A**. Matrices showing inter-regional Spearman rank correlations for Fos expression at the 1 (upper) and 36 day (lower) retention delays. Axes are numbered, and correspond to brain regions listed in **Table S1**. Colors reflect correlation strength (scale, right). **B**. Network graphs were generated by considering only the strongest correlations (Spearmans's r_s_≥0.83). In these graphs, regions are grouped by major brain subdivision and node size is proportional to the number of connections (degree) while the weight of the connection is proportional to correlation strength.
(TIF)

Figure S4 **Relationship between magnitude and variance of regional Fos signal and correlation strength.** **A**. Overall Fos signal levels (Fos^+^ nuclei/10,000 µm^2^) in WT and α-CaMKII^+/−^ mice tested at the 1 and 36 day delay. Fos levels were elevated in the WT mice that were tested at the long retention delay. **B**. Scatterplot of mean correlation strength (r^2^) vs. magnitude of Fos signal for each brain region. Data points are taken from all 4 groups tested. There was no relationship between correlation strength and the magnitude of the Fos signal, indicating that group differences in number of functional connections (or network density) cannot be attributed to overall differences in Fos expression. **C**. Overall coefficient of variation (standard deviation/mean Fos) in WT and α-CaMKII^+/−^ mice tested at the 1 and 36 day delay. Variability did not differ across groups. **D**. Scatterplot of mean correlation strength (r^2^) vs. coefficient of variation for each brain region. Data points are taken from all 4 groups tested. While correlation strength typically increased as a function of variance (or coefficient of variation), variance was equivalent across groups and therefore cannot account for increased network connectivity in WT mice at the long retention delay.
(TIF)

Figure S5 **Patterns of inter-regional correlations derived from Fos and Egr-1 expression are similar.** Matrices showing inter-regional correlations for (**A**) Fos and (**B**) Egr-1 expression in a subset of brain regions in WT mice tested at the 36 day retention delay. Colors reflect correlation strength (scale, right). Overall correlation strength did not differ in the Fos vs. Egr-1 matrices (by permutation testing; *P* = 0.76), nor were any individual inter-regional correlations different (by permutation testing; all *P*s>0.05, corrected for multiple comparisons with the false discovery rate set at 5%).
(TIF)

Figure S6 **Correspondence between structural and functional connectivity for the reuniens thalamic nucleus (Re).** Of the 84 regions investigated, 47 demonstrated direct structural (afferent or efferent) connectivity with the Re in published tract tracing studies in rodents (green circles, for a listing of all connections see **Table S2**). Regions that demonstrate functional connectivity with the Re in the high (left), primary (center) and low (right) confidence long term memory networks are represented by red circles. For the high and primary networks, all functional connections had corresponding structural connections. For the low confidence network, the majority (22/24) of functional connections had corresponding structural connections.
(TIF)

Figure S7 **Degree distribution in high and low confidence networks.** Histogram showing degree distribution for fear memory networks in WT mice tested at the 1 day (upper) and 36 day (lower) retention delay. These networks were constructed by imposing either (**A**) more (r>0.87, high confidence) or (**B**) less (r>0.79, low confidence) stringent thresholds on correlation matrices.
(TIF)

Figure S8 **Segregation in high and low confidence networks.** Mean clustering coefficient constructed by imposing (**A**) more (r>0.87, high confidence) or (**B**) less (r>0.79, low confidence) stringent thresholds on correlation matrices for WT mice tested at the 1 day (upper) and 36 day (lower) delay. The bootstrapped mean clustering coefficient for the fear memory networks are compared to the average of 1000 random networks matched for node, degree and degree distribution. Error bars represent 95% confidence intervals.
(TIF)

Figure S9 **Alternate measures of segregation for WT mice tested at the 1 and 36 day delay.** (**A**) Transitivity and (**B**) mean local efficiency measured in fear memory networks for WT mice tested at the 1 day (upper) and 36 day (lower) retention delay. The mean of bootstrapped measures in the fear memory networks are compared to the average of 1000 random networks matched for node, degree and degree distribution. Error bars represent 95% confidence intervals. These alternate measures indicate that fear memory networks are more segregated than would be expected by chance.
(TIF)

Figure S10 **Integration measures in high and low confidence networks.** Mean global efficiency in graphs constructed by imposing (**A**) more (r>0.87, high confidence) or (**B**) less (r>0.79, low confidence) stringent thresholds on correlation matrices for WT mice tested at the 1 day (upper) and 36 day (lower) delay. The bootstrapped mean global efficiency for the fear memory networks are compared to the average of 1000 random networks matched for node, degree and degree distribution. Error bars represent 95% confidence intervals.
(TIF)

Figure S11 **Alternate measure of integration for WT mice tested at the 1 and 36 day delay.** Characteristic path length for fear memory networks for WT mice tested at the 1 day (upper) and 36 day (lower) retention delay. The bootstrapped characteristic path length in the fear memory networks is compared to the average of 1000 random networks matched for node, degree and degree distribution. Error bars represent 95% confidence intervals. Fear memory and random networks had similar characteristic path length, suggesting they have equivalent levels of integration.
(TIF)

Figure S12 **Ranked centrality measures in high and low confidence networks.** Brain regions are ranked in descending order for degree and betweenness in the (**A**) high and (**B**) low confidence long term memory networks. Regions to the left of the hatched line are ranked above the 80^th^ percentile and dark colors indicate regions that are additionally ranked above 80^th^ percentile for both degree *and* betweenness. These regions were used for hub identification, shown in **Figure 7C**.
(TIF)

Figure S13 **Nodes ranked high in betweenness differ from those in random networks.** Mean betweenness values for nodes in 100 random networks matched for node, degree and degree distribution is superimposed on the ranked betweenness values in the primary (**A**), high (**B**) and low (**C**) confidence networks. High betweenness nodes differ from those derived from the random networks.
(TIF)

Figure S14 **Networks have small-world properties in α-CaMKII^+/−^ mice.** **A**. Histogram showing degree distribution for fear memory networks corresponding to 1 day (upper) and 36 day (lower) retention delay. **B**. Mean clustering coefficient for fear memory vs. random network (matched for node, degree and degree distribution). At both short (upper) and long (lower) retention delays the fear memory network was more clustered. **C**. Mean global efficiency for fear memory vs. random network. At both short (upper) and long (lower) retention delays global efficiency (or integration) was equivalent in the fear memory vs. random networks. Error bars represent 95% confidence intervals.
(TIF)

Figure S15 **Context generalization increases as a function of retention delay.** **A**. Experimental design. Mice were trained in an identical way as in the main experiment (5 footshocks in context A). Freezing was then assessed either 1 day (n = 12) or 36 days (n = 14) later in the training context (context A) and an alternate context (context B). **B**. Percent time freezing in contexts A and B at the short (1 day) vs. long (36 day) retention delay. **C**. Context discrimination ([freezing_A_−freezing_B_]/[freezing_A_+freezing_B_]) declined as a function of retention delay.
(TIF)

Table S1 **List of brain regions in which Fos was quantified with corresponding abbreviations (according to supplementary reference** [**8**] **in Text S1) and reference numbers for the correlation matrices. Brain regions are categorized by major brain subdivision.**
(PDF)

Table S2 **Direct afferent and efferent connections with the reuniens thalamic nucleus (Re).** Connections were identified from published tract tracing studies in rodents (see supplementary references [9]–[45] in **Text S1**). Brain structure from which each connection originates from and terminates are listed, together with tracer method and strength of projection (if reported).
(PDF)

Table S3 **Mean correlation coefficients within or between major brain subdivisions for WT mice tested 1 vs. 36 days after training, with corresponding 95% confidence interval of the difference.** Time-dependent changes in correlation strength were only considered significant if the 95% confidence interval of the difference was greater than zero (in red). mPFC = medial prefrontal cortex (includes Cg-a, PrL, IL and Cg-p).
(PDF)

Table S4 **Over-representation of cortical, hippocampal and thalamic regions in the densely-interconnected central component.** Using a clustering algorithm, the fear memory network (for the WT/36 day group) was organized into eight distinct clusters that included, in particular, a large densely-connected central component containing two groupings (green and blue nodes). This table reports the proportion of brain regions in major brain subdivisions with respect to the total number of regions analyzed, as well as the proportion of brain regions in major brain subdivisions with respect to the total number of regions found in the blue and green clusters. Cortical and hippocampal regions were over-represented in the green cluster, and thalamic regions were over-represented in the blue cluster (highlighted in red).
(PDF)

Table S5 **Brain regions implicated in long-term memory expression at remote time-points following training.** A pubmed-based search was conducted to identify brain regions where targeted, loss-of-function manipulations affect expression of remote memory expression (see supplementary references [46]–[73] in **Text S1**). Behavioral paradigms were considered if acquisition and/or initial memory expression (e.g., 24 hours after training) were dependent upon the hippocampus. Loss-of-function manipulations included anatomical lesions, pharmacological inactivation, DNA demethylation, and testing occurred at least 25 days after the completion of training. DNMT = DNA methyltransferases. ^a^dorsal hippocampus, ^b^ventral hippocampus, ^c^dorsal+ventral hippocampus.
(PDF)

Text S1 **Supplementary methods, results, notes and references.**
(PDF)
