# Supplementary figures and images for: Identification of a Functional Connectome for Long-Term Fear Memory in Mice
Source: PLoS Comput Biol. 2013 Jan 3;9(1):e1002853. doi: 10.1371/journal.pcbi.1002853 (PMC3536620; doi:10.1371/journal.pcbi.1002853)

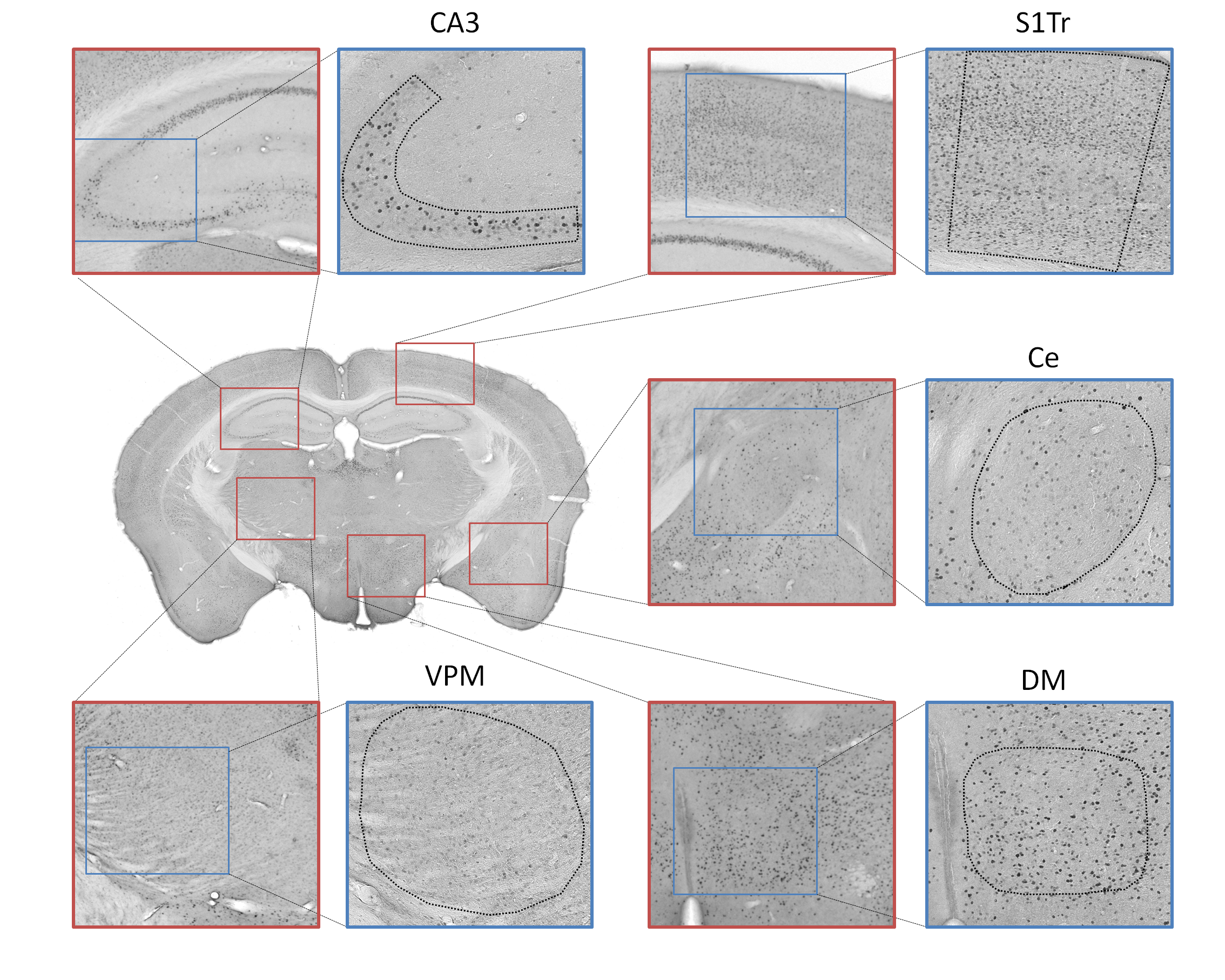

Supplement: Figure S1 — Fos quantification. Sample images of Fos immunohistochemical staining from a representative brain section. Images were captured with the 2× objective (red border) and 10× objective (blue border). Higher magnification images are shown for specific hippocampal (CA3), cortical (S1Tr), cerebral nuclei (Ce), hypothalamic (DM) and thalamic (VPM) regions. Dotted lines indicate region borders. (TIF) [file pcbi.1002853.s001.tif]

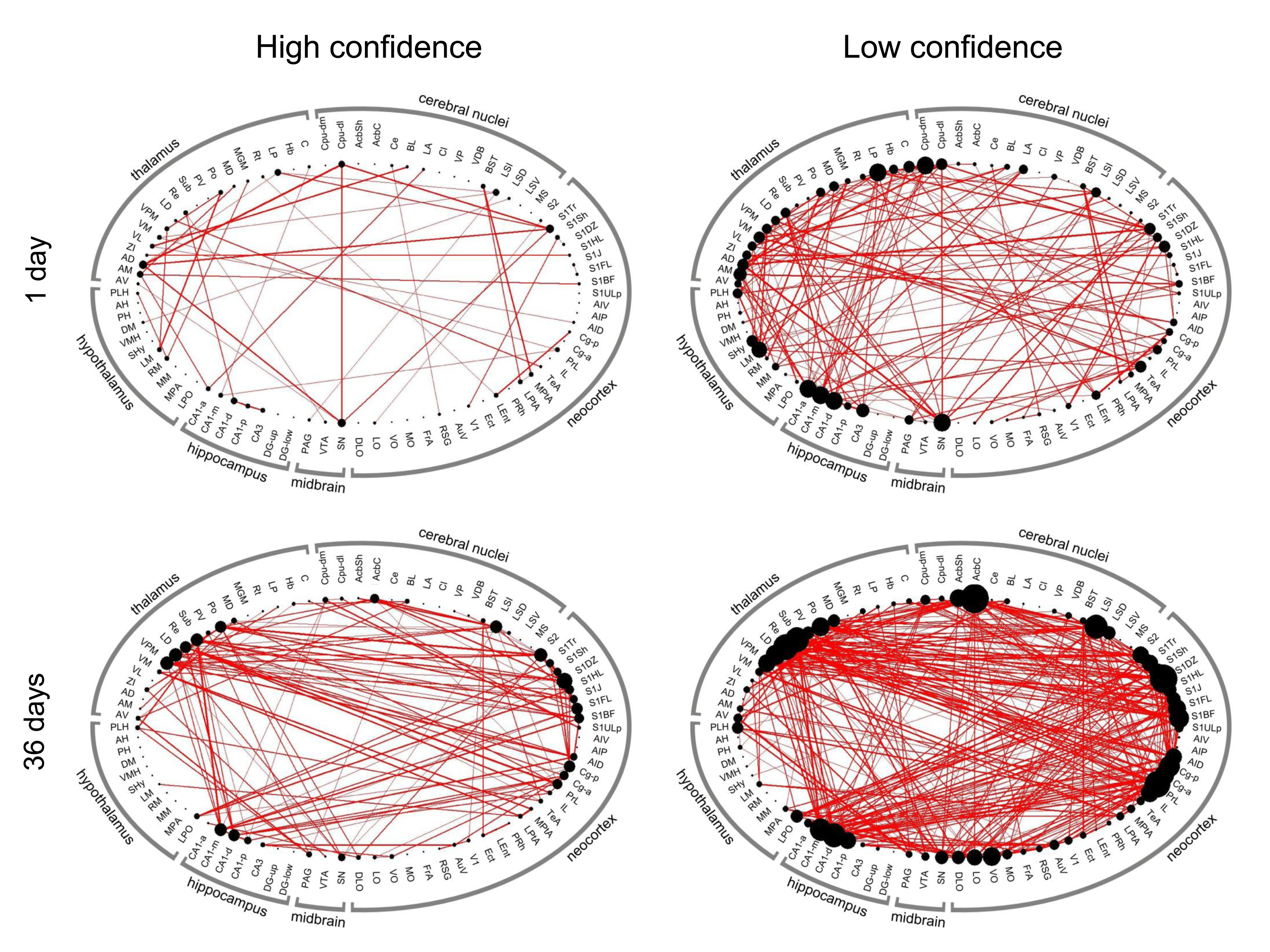

Supplement: Figure S2 — High and low confidence networks in WT mice. Networks formed from thresholding inter-regional Fos correlations in mice tested at the 1 (upper) and 36 day (lower) retention delay using either a high confidence threshold of r >0.87 (left) or a low confidence threshold of r >0.79 (right). Brain regions are grouped by major brain subdivision and node size is proportional to the number of connections (degree) while the weight of the connection is proportional to correlation strength. (TIF) [file pcbi.1002853.s002.tif]

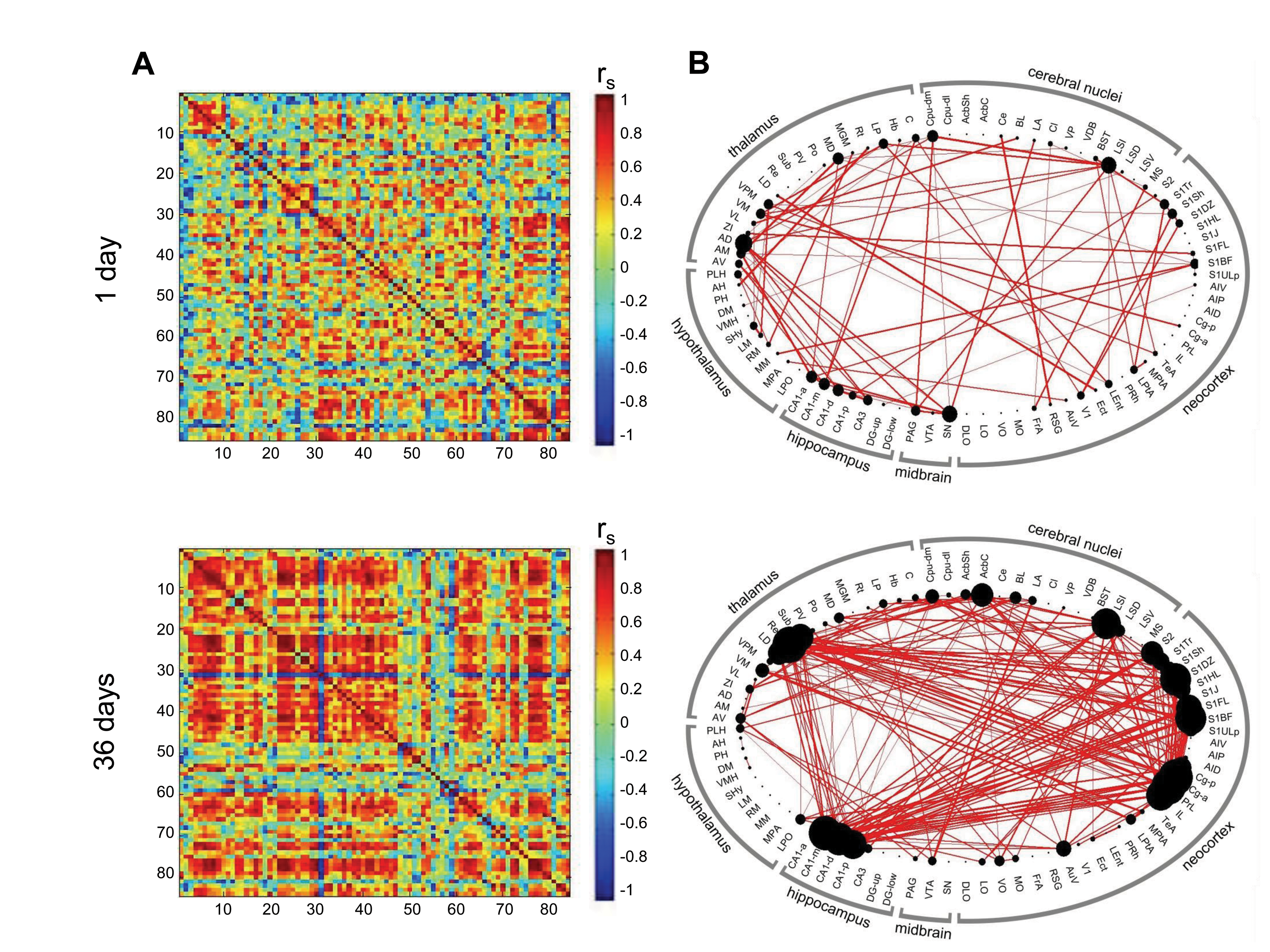

Supplement: Figure S3 — Spearman rank correlation networks in WT mice. A. Matrices showing inter-regional Spearman rank correlations for Fos expression at the 1 (upper) and 36 day (lower) retention delays. Axes are numbered, and correspond to brain regions listed in Table S1. Colors reflect correlation strength (scale, right). B. Network graphs were generated by considering only the strongest correlations (Spearmans's rs≥0.83). In these graphs, regions are grouped by major brain subdivision and node size is proportional to the number of connections (degree) while the weight of the connection is proportional to correlation strength. (TIF) [file pcbi.1002853.s003.tif]

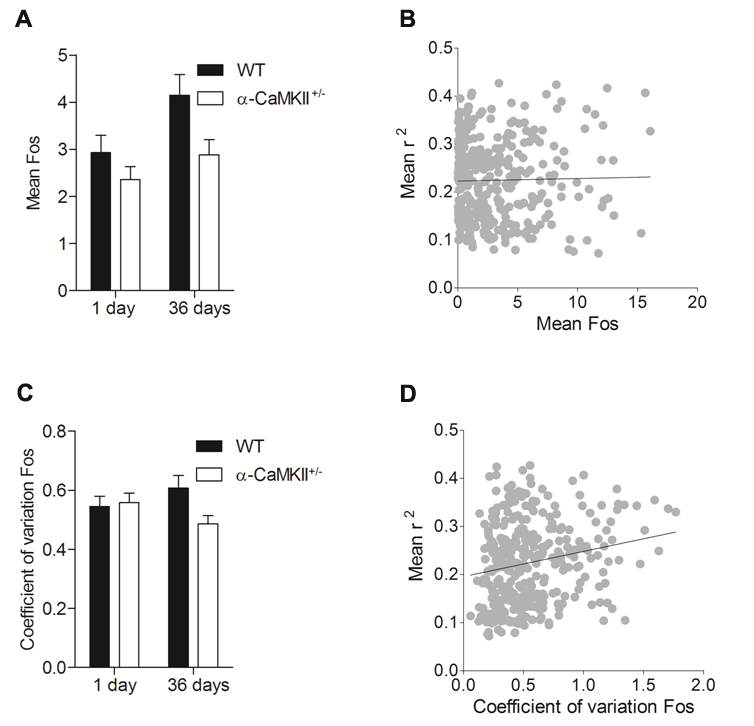

Supplement: Figure S4 — Relationship between magnitude and variance of regional Fos signal and correlation strength. A. Overall Fos signal levels (Fos+ nuclei/10,000 µm2) in WT and α-CaMKII+/− mice tested at the 1 and 36 day delay. Fos levels were elevated in the WT mice that were tested at the long retention delay. B. Scatterplot of mean correlation strength (r2) vs. magnitude of Fos signal for each brain region. Data points are taken from all 4 groups tested. There was no relationship between correlation strength and the magnitude of the Fos signal, indicating that group differences in number of functional connections (or network density) cannot be attributed to overall differences in Fos expression. C. Overall coefficient of variation (standard deviation/mean Fos) in WT and α-CaMKII+/− mice tested at the 1 and 36 day delay. Variability did not differ across groups. D. Scatterplot of mean correlation strength (r2) vs. coefficient of variation for each brain region. Data points are taken from all 4 groups tested. While correlation strength typically increased as a function of variance (or coefficient of variation), variance was equivalent across groups and therefore cannot account for increased network connectivity in WT mice at the long retention delay. (TIF) [file pcbi.1002853.s004.tif]

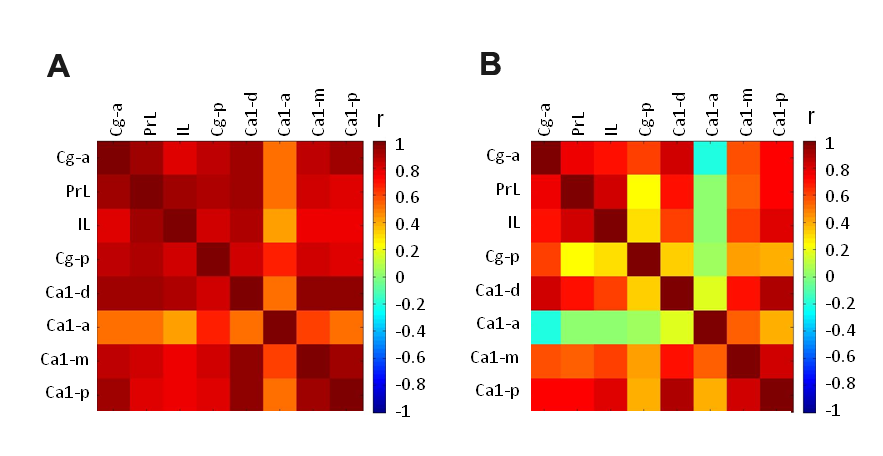

Supplement: Figure S5 — Patterns of inter-regional correlations derived from Fos and Egr-1 expression are similar. Matrices showing inter-regional correlations for (A) Fos and (B) Egr-1 expression in a subset of brain regions in WT mice tested at the 36 day retention delay. Colors reflect correlation strength (scale, right). Overall correlation strength did not differ in the Fos vs. Egr-1 matrices (by permutation testing; P = 0.76), nor were any individual inter-regional correlations different (by permutation testing; all Ps>0.05, corrected for multiple comparisons with the false discovery rate set at 5%). (TIF) [file pcbi.1002853.s005.tif]

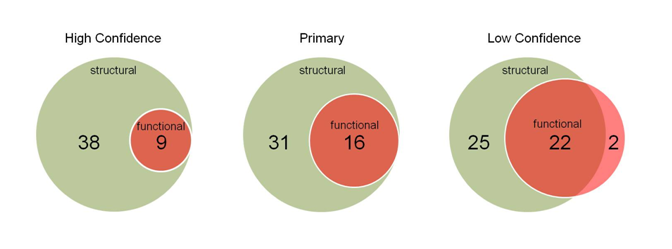

Supplement: Figure S6 — Correspondence between structural and functional connectivity for the reuniens thalamic nucleus (Re). Of the 84 regions investigated, 47 demonstrated direct structural (afferent or efferent) connectivity with the Re in published tract tracing studies in rodents (green circles, for a listing of all connections see Table S2). Regions that demonstrate functional connectivity with the Re in the high (left), primary (center) and low (right) confidence long term memory networks are represented by red circles. For the high and primary networks, all functional connections had corresponding structural connections. For the low confidence network, the majority (22/24) of functional connections had corresponding structural connections. (TIF) [file pcbi.1002853.s006.tif]

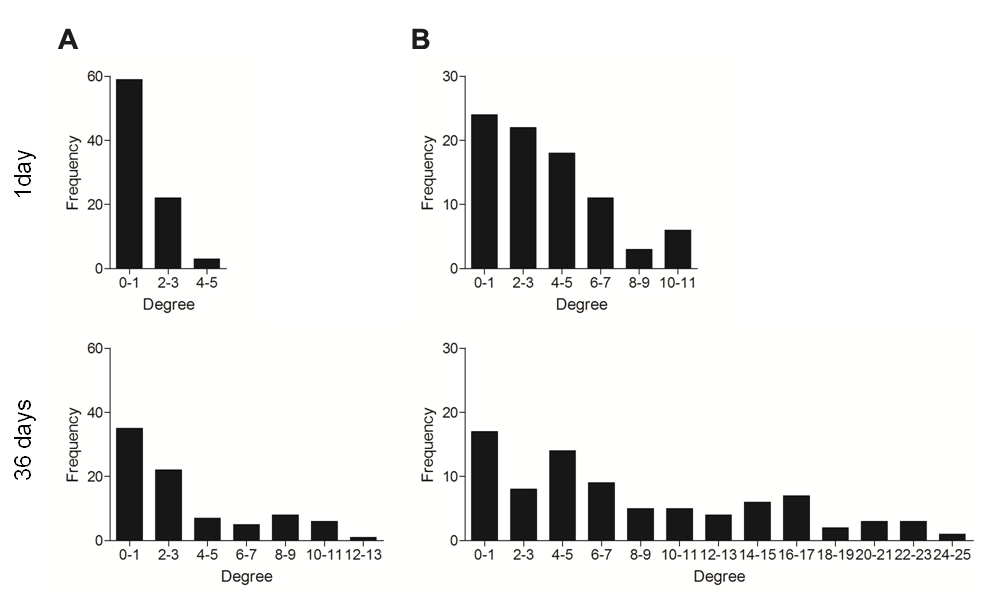

Supplement: Figure S7 — Degree distribution in high and low confidence networks. Histogram showing degree distribution for fear memory networks in WT mice tested at the 1 day (upper) and 36 day (lower) retention delay. These networks were constructed by imposing either (A) more (r>0.87, high confidence) or (B) less (r>0.79, low confidence) stringent thresholds on correlation matrices. (TIF) [file pcbi.1002853.s007.tif]

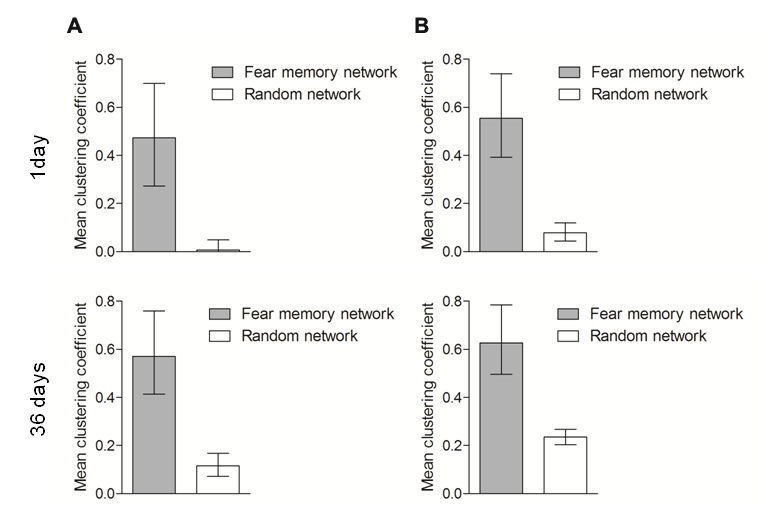

Supplement: Figure S8 — Segregation in high and low confidence networks. Mean clustering coefficient constructed by imposing (A) more (r>0.87, high confidence) or (B) less (r>0.79, low confidence) stringent thresholds on correlation matrices for WT mice tested at the 1 day (upper) and 36 day (lower) delay. The bootstrapped mean clustering coefficient for the fear memory networks are compared to the average of 1000 random networks matched for node, degree and degree distribution. Error bars represent 95% confidence intervals. (TIF) [file pcbi.1002853.s008.tif]

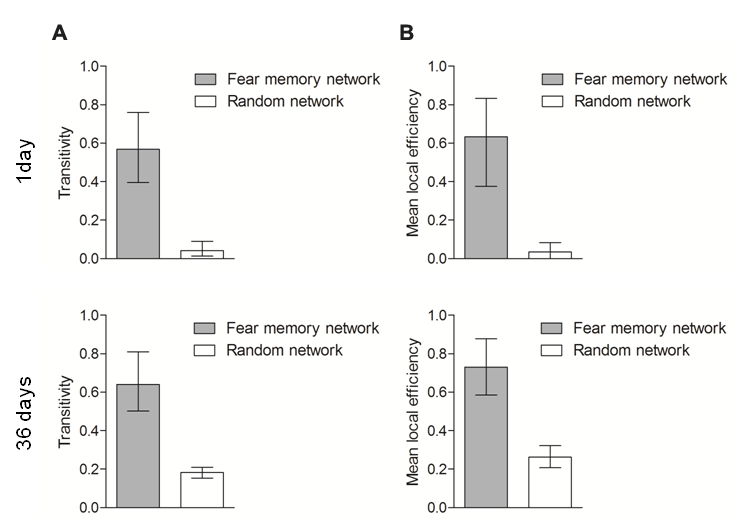

Supplement: Figure S9 — Alternate measures of segregation for WT mice tested at the 1 and 36 day delay. (A) Transitivity and (B) mean local efficiency measured in fear memory networks for WT mice tested at the 1 day (upper) and 36 day (lower) retention delay. The mean of bootstrapped measures in the fear memory networks are compared to the average of 1000 random networks matched for node, degree and degree distribution. Error bars represent 95% confidence intervals. These alternate measures indicate that fear memory networks are more segregated than would be expected by chance. (TIF) [file pcbi.1002853.s009.tif]

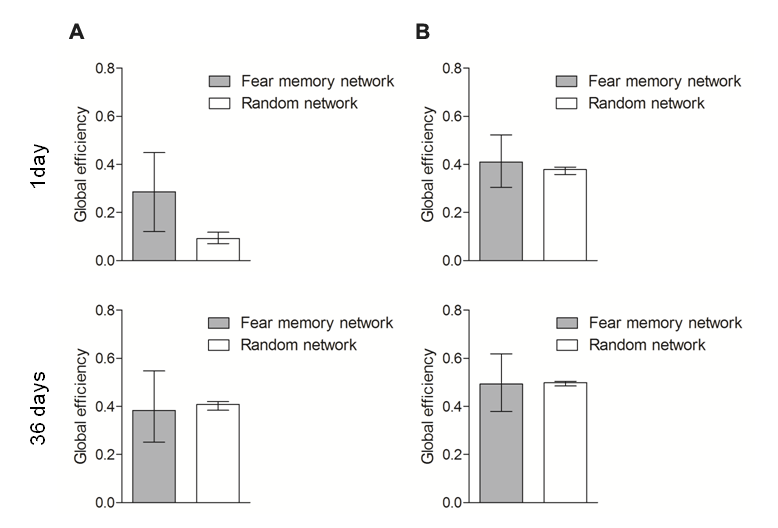

Supplement: Figure S10 — Integration measures in high and low confidence networks. Mean global efficiency in graphs constructed by imposing (A) more (r>0.87, high confidence) or (B) less (r>0.79, low confidence) stringent thresholds on correlation matrices for WT mice tested at the 1 day (upper) and 36 day (lower) delay. The bootstrapped mean global efficiency for the fear memory networks are compared to the average of 1000 random networks matched for node, degree and degree distribution. Error bars represent 95% confidence intervals. (TIF) [file pcbi.1002853.s010.tif]

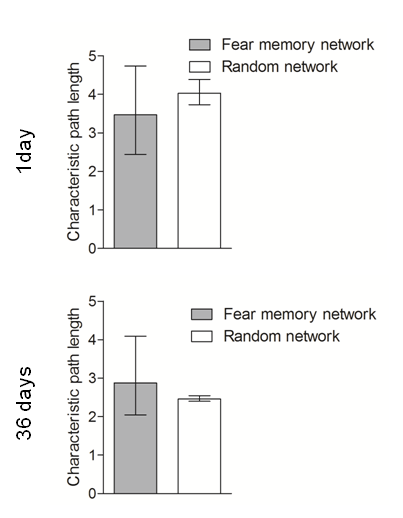

Supplement: Figure S11 — Alternate measure of integration for WT mice tested at the 1 and 36 day delay. Characteristic path length for fear memory networks for WT mice tested at the 1 day (upper) and 36 day (lower) retention delay. The bootstrapped characteristic path length in the fear memory networks is compared to the average of 1000 random networks matched for node, degree and degree distribution. Error bars represent 95% confidence intervals. Fear memory and random networks had similar characteristic path length, suggesting they have equivalent levels of integration. (TIF) [file pcbi.1002853.s011.tif]

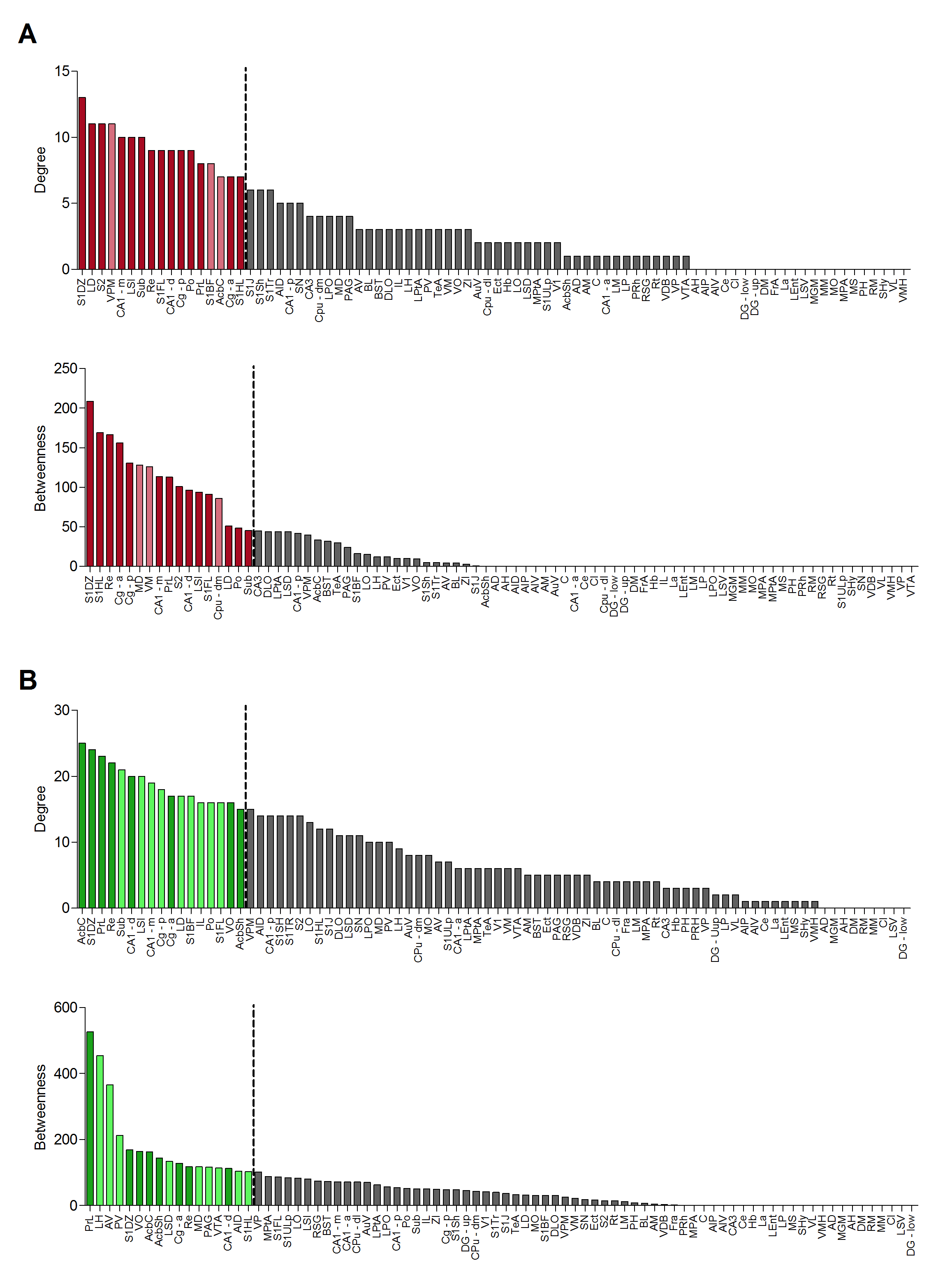

Supplement: Figure S12 — Ranked centrality measures in high and low confidence networks. Brain regions are ranked in descending order for degree and betweenness in the (A) high and (B) low confidence long term memory networks. Regions to the left of the hatched line are ranked above the 80th percentile and dark colors indicate regions that are additionally ranked above 80th percentile for both degree and betweenness. These regions were used for hub identification, shown in Figure 7C . (TIF) [file pcbi.1002853.s012.tif]

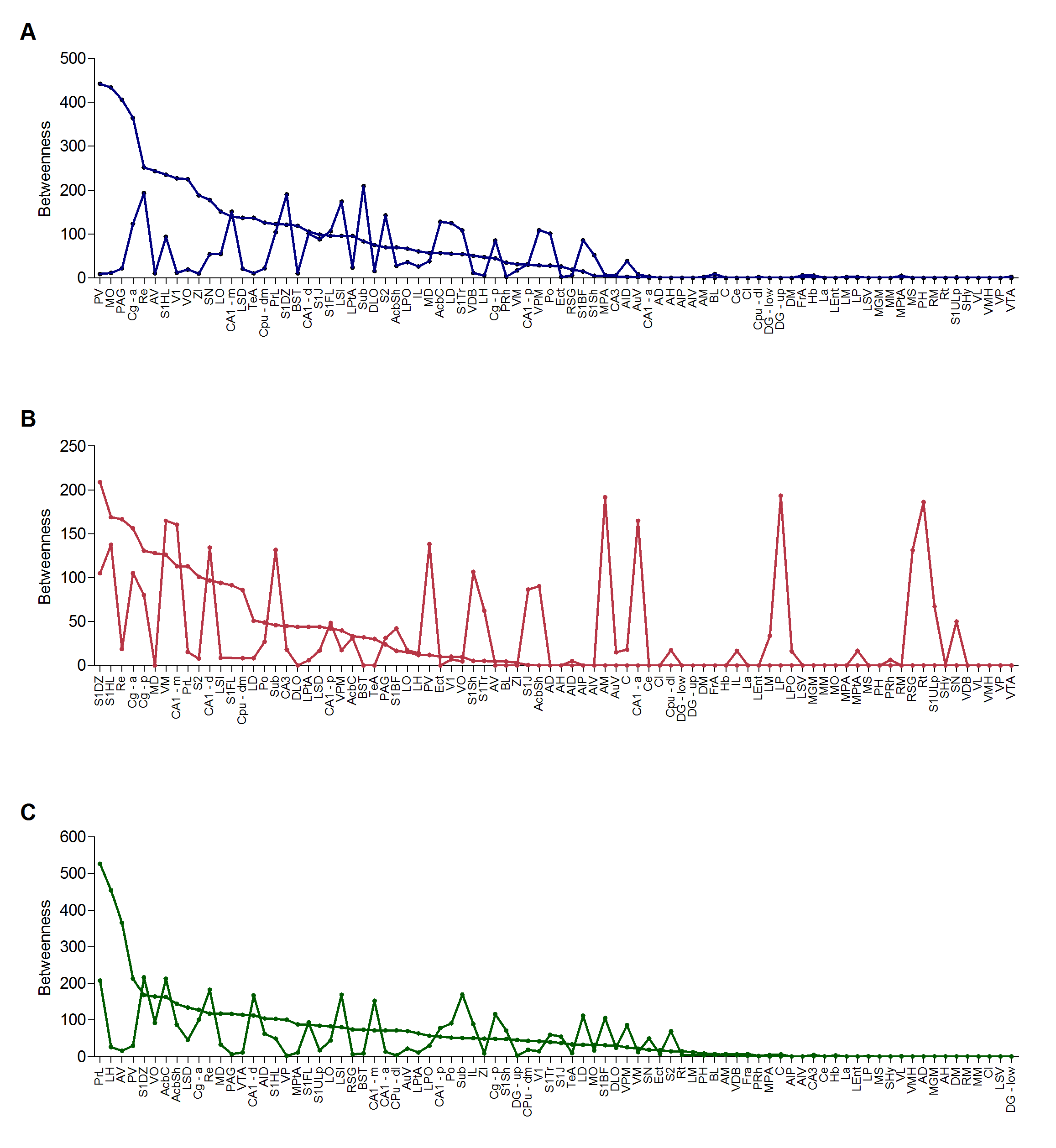

Supplement: Figure S13 — Nodes ranked high in betweenness differ from those in random networks. Mean betweenness values for nodes in 100 random networks matched for node, degree and degree distribution is superimposed on the ranked betweenness values in the primary (A), high (B) and low (C) confidence networks. High betweenness nodes differ from those derived from the random networks. (TIF) [file pcbi.1002853.s013.tif]

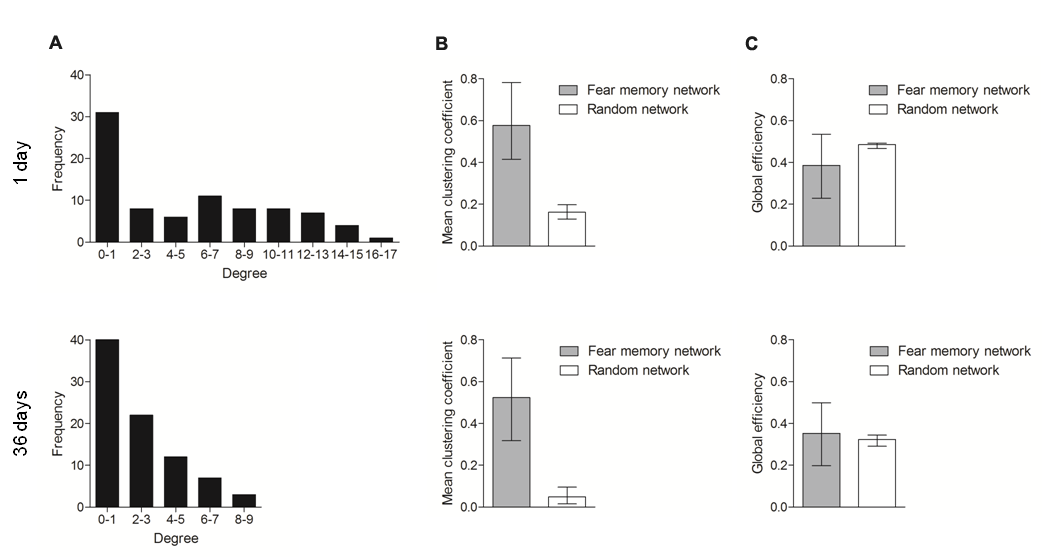

Supplement: Figure S14 — Networks have small-world properties in α-CaMKII+/− mice. A. Histogram showing degree distribution for fear memory networks corresponding to 1 day (upper) and 36 day (lower) retention delay. B. Mean clustering coefficient for fear memory vs. random network (matched for node, degree and degree distribution). At both short (upper) and long (lower) retention delays the fear memory network was more clustered. C. Mean global efficiency for fear memory vs. random network. At both short (upper) and long (lower) retention delays global efficiency (or integration) was equivalent in the fear memory vs. random networks. Error bars represent 95% confidence intervals. (TIF) [file pcbi.1002853.s014.tif]

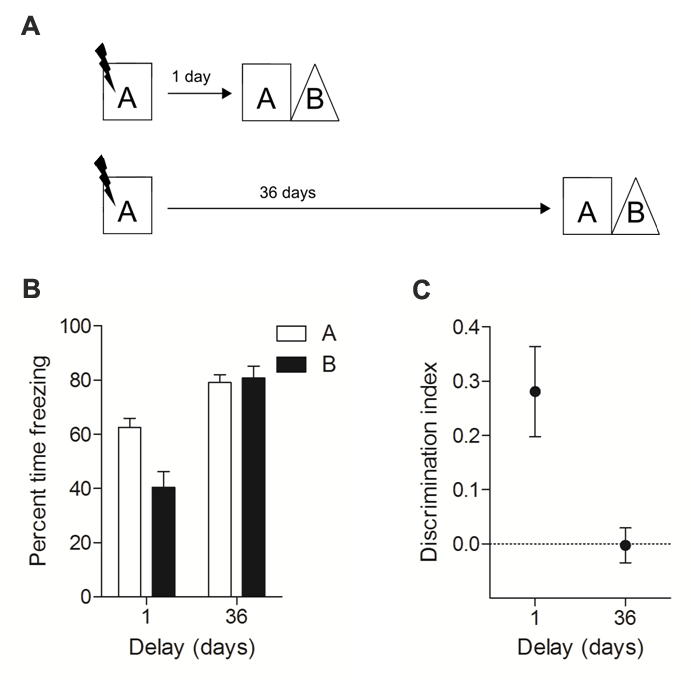

Supplement: Figure S15 — Context generalization increases as a function of retention delay. A. Experimental design. Mice were trained in an identical way as in the main experiment (5 footshocks in context A). Freezing was then assessed either 1 day (n = 12) or 36 days (n = 14) later in the training context (context A) and an alternate context (context B). B. Percent time freezing in contexts A and B at the short (1 day) vs. long (36 day) retention delay. C. Context discrimination ([freezingA−freezingB]/[freezingA+freezingB]) declined as a function of retention delay. (TIF) [file pcbi.1002853.s015.tif]
