# Supplementary material for: Identification of a Functional Connectome for Long-Term Fear Memory in Mice
Source: PLoS Comput Biol. 2013 Jan 3;9(1):e1002853. doi: 10.1371/journal.pcbi.1002853 (PMC3536620; doi:10.1371/journal.pcbi.1002853)
Supplement: Table S1 — List of brain regions in which Fos was quantified with corresponding abbreviations (according to supplementary reference [8] in Text S1) and reference numbers for the correlation matrices. Brain regions are categorized by major brain subdivision. (PDF) [file pcbi.1002853.s016.pdf]

Region	Abbreviation	#
**Neocortex**		
frontal association cortex	FrA	1
dorsolateral orbital cortex	DLO	2
lateral orbital cortex	LO	3
ventral orbital cortex	VO	4
medial orbital cortex	MO	5
infralimbic cortex	IL	6
prelimbic cortex	PrL	7
cingulate cortex - anteror	Cg-a	8
cingulate cortex - posterior	Cg-p	9
retrosplenial granular cortex	RSG	10
ectorhinal cortex	Ect	11
lateral entorhinal cortex	LEnt	12
lateral parietal association cortex	LPtA	13
medial parietal association cortex	MPtA	14
perirhinal cortex	PRh	15
temporal association cortex	TeA	16
secondary auditory cortex, ventral area	AuV	17
primary visual cortex	V1	18
agranular insular cortex, posterior part	AIP	19
agranular insular cortex, dorsal part	AID	20
agranular insular cortex, ventral part	AIV	21
primary somatosensory cortex, barrel field	S1BF	22
primary somatosensory cortex, dysgranular region	S1DZ	23
primary somatosensory cortex, forelimb region	S1FL	24
primary somatosensory cortex, hindlimb region	S1HL	25
primary somatosensory cortex, jaw region	S1J	26
primary somatosensory cortex, shoulder region	S1Sh	27
primary somatosensory cortex, trunk region	S1Tr	28
primary somatosensory cortex, upper lip region	S1ULp	29
secondary somatosensory cortex	S2	30
**Thalamus**		
anterodorsal thalamic nucleus	AD	31
anteromedial thalamic nucleus	AM	32
anteroventral thalamic nucleus	AV	33
central thalamic nucleus	C	34
habenular nucleus	Hb	35
laterodorsal thalamic nucleus	LD	36
lateral posterior thalamic nucleus	LP	37
mediodorsal thalamic nucleus	MD	38
posterior thalamic nuclear group	Po	39
paraventricular thalamic nucleus	PV	40
reuniens thalamic nucleus	Re	41
submedius thalamic nucleus	Sub	42
ventrolateral thalamic nucleus	VL	43
ventromedial thalamic nucleus	VM	44

Region	Abbreviation	#
**Thalamus (cont.)**		
ventral posteromedial thalamic nucleus	VPM	45
reticular thalamic nucleus	Rt	46
zona inserta	ZI	47
medial geniculate nucleus, medial part	MGM	48
**Hypothalamus**		
anterior hypothalamic area	AH	49
posterior hypothalamic area	PH	50
dorsomedial hypothalamic nucleus	DM	51
ventromedial hypothalamic nucleus	VMH	52
lateral hypothalamic area	LH	53
lateral preoptic area	LPO	54
medial preoptic area	MPA	55
septohypothalamic nucleus	SHy	56
lateral mammilary nucleus	LM	57
retromammilary nucleus	RM	58
medial mammilary nucleus	MM	59
**Cerebral nuclei**		
bed nucleus of the stria terminalis	BST	60
caudate putamen (striatum) - dorsal lateral	Cpu-dl	61
caudate putamen (striatum) - dorsal medial	Cpu-dm	62
accumbens nucleus, core	AcbC	63
accumbens nucleus, shell	AcbSh	64
ventral pallidum	VP	65
nucleus of the vertical limb of the diagonal band	VDB	66
claustrum	Cl	67
lateral amygdaloid nucleus	La	68
central amygdaloid nucleus	Ce	69
basolateral amygdaloid nucleus	BL	70
medial septal nucleus	MS	71
lateral septal nucleus, dorsal part	LSD	72
lateral septal nucleus, intermediate part	LSI	73
lateral septal nucleus, ventral part	LSV	74
**Hippocampus**		
field CA1 of hippocampus - anterior	CA1-a	75
field CA1 of hippocampus - medial	CA1-m	76
field CA1 of hippocampus - distal	CA1-d	77
field CA1 of hippocampus - posterior	CA1-p	78
field CA3 of hippocampus	CA3	79
dentate gyrus - upper blade	DG-up	80
dentate gyrus - lower blade	DG-low	81
**Midbrain**		
ventral tegmental area	VTA	82
periaqueductal gray	PAG	83
substantia nigra	SN	84
