# Supplementary material for: Identification of a Functional Connectome for Long-Term Fear Memory in Mice
Source: PLoS Comput Biol. 2013 Jan 3;9(1):e1002853. doi: 10.1371/journal.pcbi.1002853 (PMC3536620; doi:10.1371/journal.pcbi.1002853)
Supplement: Table S2 — Direct afferent and efferent connections with the reuniens thalamic nucleus (Re). Connections were identified from published tract tracing studies in rodents (see supplementary references [9]–[45] in Text S1). Brain structure from which each connection originates from and terminates are listed, together with tracer method and strength of projection (if reported). (PDF) [file pcbi.1002853.s017.pdf]

From	To	Method	Projection strength	Reference
AH	Re	Fluorogold	moderate	(McKenna & Vertes 2004)
AH	Re	PHA-L	exists	(Risold et al. 1994)
AH	Re	Fluorogold	exists	(Risold & Swanson 1995)
AH	Re	Fluorogold	exists	(Risold & Swanson 1995)
AH	Re	PHA-L	exists	(Risold et al. 1997)
AID	Re	Fluorogold	moderate	(McKenna & Vertes 2004)
AIP	Re	HRP	light	(Deacon et al. 1983)
AIP	Re	Fluorogold	light	(McKenna & Vertes 2004)
AIV	Re	Fluorogold	light	(McKenna & Vertes 2004)
BL	Re	PHA-L	very light	(Petrovich et al. 1996)
BST	Re	PHA-L	exists	(Dong & Swanson 2006a)
BST	Re	PHA-L	exists	(Dong & Swanson 2006b)
BST	Re	PHA-L	light	(Dong & Swanson 2006c)
BST	Re	PHA-L	exists	(Dong & Swanson 2004b)
BST	Re	PHA-L	exists	(Dong & Swanson 2004a)
BST	Re	PHA-L	exists	(Dong et al. 2001)
BST	Re	Fluorogold	light	(McKenna & Vertes 2004)
CA1	Re	PHA-L	very light	(Cenquizca & Swanson 2006)
CA1	Re	PHA-L	very light	(Cenquizca & Swanson 2006)
CA1	Re	PHA-L	very light	(Cenquizca & Swanson 2006)
Cg-a	Re	autoradiography	light	(Beckstead 1979)
Cg-a	Re	HRP/WGA	light	(Finch et al. 1984)
Cg-a	Re	Fluorogold	moderate	(McKenna & Vertes 2004)
Cg-a	Re	PHA-L	exists	(Risold et al. 1997)
Cl	Re	Fluorogold	strong	(McKenna & Vertes 2004)
Cl	Re	PHA-L	exists	(Risold et al. 1997)
DM	Re	Fluorogold	light	(McKenna & Vertes 2004)
DM	Re	Fluorogold	exists	(Risold & Swanson 1995)
DM	Re	PHA-L	exists	(Thompson et al. 1996)
Ect	Re	HRP	moderate	(Deacon et al. 1983)
Ect	Re	Fluorogold	light	(McKenna & Vertes 2004)
Hb	Re	Fluorogold	moderate	(McKenna & Vertes 2004)
Hippocampus	Re	fluorescent tracers	exists	(Woolf et al. 1984)
IL	Re	fluorescent tracers	moderate	(Freedman & Cassell 1991)
IL	Re	Fluorogold	strong	(McKenna & Vertes 2004)
IL	Re	PHA-L	exists	(Risold et al. 1997)
LH	Re	PHA-L	light	(Allen & Cechetto 1993)
LH	Re	PHA-L	light/moderate	(Goto et al. 2005)
LH	Re	Fluorogold	strong	(McKenna & Vertes 2004)
LH	Re	PHA-L	exists	(Risold et al. 1994)
LH	Re	Fluorogold	exists	(Risold & Swanson 1995)

LM	Re	Fluorogold	moderate	(McKenna & Vertes 2004)
LPO	Re	PHA-L	exists	(Risold et al. 1997)
LS	Re	Fluorogold	moderate	(McKenna & Vertes 2004)
LSV	Re	PHA-L	exists	(Risold & Swanson 1997)
MM	Re	PHA-L	exists	(Canteras & Swanson 1992)
MM	Re	Fluorogold	light	(McKenna & Vertes 2004)
MO	Re	Fluorogold	strong	(McKenna & Vertes 2004)
MPO	Re	PHA-L	exists	(Simerly & Swanson 1988)
PAG	Re	Fluorogold	moderate	(McKenna & Vertes 2004)
PAG	Re	PHA-L	exists	(Risold et al. 1997)
PH	Re	Fluorogold	strong	(McKenna & Vertes 2004)
PH	Re	Fluorogold	exists	(Risold & Swanson 1995)
PH	Re	PHA-L	exists	(Risold et al. 1997)
PrH	Re	HRP	moderate	(Deacon et al. 1983)
PrH	Re	Fluorogold	light	(McKenna & Vertes 2004)
PrH	Re	PHA-L	exists	(Risold et al. 1997)
PrL	Re	autoradiography	light	(Beckstead 1979)
PrL	Re	PHA-L	exists	(Hurley et al. 1991)
PrL	Re	Fluorogold	strong	(McKenna & Vertes 2004)
PrL	Re	PHA-L	exists	(Risold et al. 1997)
PrL	Re	PHA-L	moderate	(Sesack et al. 1989)
PrL	Re	HRP/WGA	light	(Zeng & Stuesse 1991)
PV	Re	Fluorogold	exists	(Risold & Swanson 1995)
PV	Re	PHA-L	exists	(Thompson & Swanson 2003)
RSG	Re	HRP/WGA	light	(Finch et al. 1984)
RSG	Re	Fluorogold	moderate	(McKenna & Vertes 2004)
RSG	Re	PHA-L	exists	(Risold & Swanson 1995)
RSG	Re	fluorescent tracers	moderate	(Sripanidkulchai & Wyss 1986)
RSG	Re	HRP	light	(Thompson & Robertson 1987)
RSG	Re	fluorescent tracers	light	(van Groen & Wyss 1990)
RSG	Re	PHA-L	light	(van Groen & Wyss 1990)
Rt	Re	Fluorogold	light	(McKenna & Vertes 2004)
Rt	Re	PHA-L	exists	(Risold et al. 1997)
S1	Re	Fluorogold	light	(McKenna & Vertes 2004)
S2	Re	Fluorogold	light	(McKenna & Vertes 2004)
TeA	Re	HRP/WGA	exists	(Arnault & Roger 1990)
V1	Re	fluorescent tracers	light	(Dreher et al. 1990)
VMH	Re	PHA-L	exists	(Canteras et al. 1994)
VMH	Re	Fluorogold	moderate	(McKenna & Vertes 2004)
VMH	Re	Fluorogold	exists	(Risold & Swanson 1995)
VTA	Re	autoradiography	light	(Beckstead et al. 1979)
VTA	Re	Fluorogold	moderate	(McKenna & Vertes 2004)

ZI	Re	Fluorogold	strong	(McKenna & Vertes 2004)
ZI	Re	PHA-L	exists	(Risold et al. 1997)
Re	Acb	PHA-L	exists	(Risold et al. 1997)
Re	AcbC	PHA-L	light	(Vertes et al. 2006)
Re	AcbS	PHA-L	light	(Vertes et al. 2006)
Re	AID	PHA-L	moderate	(Vertes et al. 2006)
Re	AIP	PHA-L	light	(Vertes et al. 2006)
Re	AIV	PHA-L	moderate	(Vertes et al. 2006)
Re	AM	PHA-L	light	(Vertes et al. 2006)
Re	AM	PHA-L	light	(Vertes et al. 2006)
Re	AV	PHA-L	light	(Vertes et al. 2006)
Re	BL	PHA-L	light	(Vertes et al. 2006)
Re	BST	PHA-L	light	(Vertes et al. 2006)
Re	BST	PHA-L	exists	(Risold et al. 1997)
Re	BST	CTB	light	(Shin et al. 2008)
Re	BST	CTB	light	(Shin et al. 2008)
Re	C	PHA-L	light	(Vertes et al. 2006)
Re	CA1	PHA-L	exists	(Risold & Swanson 1995)
Re	CA1	PHA-L	exists	(Risold et al. 1997)
Re	CA1	PHA-L	strong	(Vertes et al. 2006)
Re	Cg-a	HRP/WGA	light	(Hurley et al. 1991)
Re	Cg-a	PHA-L	exists	(Risold & Swanson 1995)
Re	Cg-a	PHA-L	strong	(Risold et al. 1997)
Re	Cg-a	PHA-L	moderate	(Vertes et al. 2006)
Re	Cl	HRP/WGA	moderate	(Hurley et al. 1991)
Re	Cl	PHA-L	strong	(Vertes et al. 2006)
Re	Cpu	PHA-L	light	(Vertes et al. 2006)
Re	Ent-l	PHA-L	exists	(Risold & Swanson 1995)
Re	Ent-l	PHA-L	exists	(Risold et al. 1997)
Re	Ent-m	PHA-L	exists	(Risold & Swanson 1995)
Re	Ent-m	PHA-L	strong	(Risold et al. 1997)
Re	FrA	PHA-L	light	(Vertes et al. 2006)
Re	Hb	PHA-L	light	(Vertes et al. 2006)
Re	Hippocampus	HRP	exists	(Wyss et al. 1979)
Re	IL	PHA-L	exists	(Risold & Swanson 1995)
Re	IL	PHA-L	strong	(Vertes et al. 2006)
Re	LA	PHA-L	exists	(Risold et al. 1997)
Re	LA	PHA-L	light	(Vertes et al. 2006)
Re	LEnt	PHA-L	strong	(Vertes et al. 2006)
Re	LO	PHA-L	moderate	(Vertes et al. 2006)
Re	LS	PHA-L	exists	(Risold et al. 1997)
Re	LSI	PHA-L	light	(Vertes et al. 2006)

Re	LSV	Fluorogold	exists	(Risold & Swanson 1997)
Re	LSV	PHA-L	light	(Vertes et al. 2006)
Re	MD	PHA-L	light	(Vertes et al. 2006)
Re	MM	PHA-L	light	(Vertes et al. 2006)
Re	MO	PHA-L	strong	(Vertes et al. 2006)
Re	MS	PHA-L	light	(Vertes et al. 2006)
Re	PL	PHA-L	strong	(Risold et al. 1997)
Re	PRh	PHA-L	exists	(Risold & Swanson 1995)
Re	PrH	PHA-L	strong	(Vertes et al. 2006)
Re	PrL	HRP/WGA	light	(Hurley et al. 1991)
Re	PrL	PHA-L	exists	(Risold & Swanson 1995)
Re	PrL	PHA-L	strong	(Vertes et al. 2006)
Re	PrL	HRP/WGA	exists	(Zeng & Stuesse 1991)
Re	PV	PHA-L	light	(Vertes et al. 2006)
Re	RSG	PHA-L	strong	(Vertes et al. 2006)
Re	RSG	Fluorogold	exists	(Risold & Swanson 1995)
Re	RSG	PHA-L	exists	(Risold & Swanson 1995)
Re	Rt	PHA-L	light	(Vertes et al. 2006)
Re	S1	PHA-L	light	(Vertes et al. 2006)
Re	S2	PHA-L	light	(Vertes et al. 2006)
Re	Sub	PHA-L	light	(Vertes et al. 2006)
Re	TeA	PHA-L	light	(Vertes et al. 2006)
Re	VDB	PHA-L	light	(Vertes et al. 2006)
Re	VL	PHA-L	light	(Vertes et al. 2006)
Re	VLO	PHA-L	light	(Vertes et al. 2006)
Re	VO	PHA-L	strong	(Vertes et al. 2006)
Re	VO	PHA-L	exists	(Risold et al. 1997)
Re	ZI	PHA-L	light	(Vertes et al. 2006)
