# Supplementary material for: Identification of a Functional Connectome for Long-Term Fear Memory in Mice
Source: PLoS Comput Biol. 2013 Jan 3;9(1):e1002853. doi: 10.1371/journal.pcbi.1002853 (PMC3536620; doi:10.1371/journal.pcbi.1002853)
Supplement: Table S3 — Mean correlation coefficients within or between major brain subdivisions for WT mice tested 1 vs. 36 days after training, with corresponding 95% confidence interval of the difference. Time-dependent changes in correlation strength were only considered significant if the 95% confidence interval of the difference was greater than zero (in red). mPFC = medial prefrontal cortex (includes Cg-a, PrL, IL and Cg-p). (PDF) [file pcbi.1002853.s018.pdf]

	1 day	36 days	Difference [95% CI]	
interneocortical	0.127	0.551	0.424	**[0.0005 0.6856]**
intersomatosensory	0.279	0.752	0.473	**[0.0499 0.7903]**
mPFC-neocortex,thalamus,hippocampus	0.312	0.603	0.292	**[0.0069 0.5782]**
mPFC-neocortex	0.268	0.669	0.401	**[0.0186 0.6481]**
mPFC-thalamus	0.432	0.551	0.118	[-0.2104 0.4889]
mPFC-hypothalamus	0.192	0.276	0.084	[-0.4080 0.4947]
mPFC-cerebral nuclei	0.285	0.501	0.216	[-0.0610 0.4608]
mPFC-hippocampus	0.325	0.575	0.250	[-0.0807 0.7248]
mPFC-midbrain	0.395	0.561	0.166	[-0.6172 0.7352]
hippocampus-neocortex	0.142	0.452	0.310	**[0.0037 0.5432]**
hippocampus-thalamus	0.305	0.373	0.068	[-0.1321 0.2799]
hippocampus-hypothalamus	0.127	0.098	-0.030	[-0.2932 0.1883]
hippocampus-cerebral nuclei	0.208	0.335	0.126	[-0.1153 0.3788]
hippocampus-hippocampus	0.303	0.448	0.144	[-0.1816 0.4183]
hippocampus-midbrain	0.458	0.372	-0.085	[-0.5344 0.1539]
