# Supplementary material for: Identification of a Functional Connectome for Long-Term Fear Memory in Mice
Source: PLoS Comput Biol. 2013 Jan 3;9(1):e1002853. doi: 10.1371/journal.pcbi.1002853 (PMC3536620; doi:10.1371/journal.pcbi.1002853)
Supplement: Table S4 — Over-representation of cortical, hippocampal and thalamic regions in the densely-interconnected central component. Using a clustering algorithm, the fear memory network (for the WT/36 day group) was organized into eight distinct clusters that included, in particular, a large densely-connected central component containing two groupings (green and blue nodes). This table reports the proportion of brain regions in major brain subdivisions with respect to the total number of regions analyzed, as well as the proportion of brain regions in major brain subdivisions with respect to the total number of regions found in the blue and green clusters. Cortical and hippocampal regions were over-represented in the green cluster, and thalamic regions were over-represented in the blue cluster (highlighted in red). (PDF) [file pcbi.1002853.s019.pdf]

| | Neocortex | Thalamus | Hypothalamus | Cerebral nuclei | Hippocampus | Midbrain |
|---|---|---|---|---|---|---|
| **Total** | **0.36** | **0.21** | 0.13 | 0.18 | **0.08** | 0.04 |
| **Blue cluster** | 0.39 | **0.30** | 0.04 | 0.17 | 0.04 | 0.04 |
| **Green cluster** | **0.58** | 0.08 | 0.00 | 0.00 | **0.33** | 0.00 |
