# Supplementary material for: Identification of a Functional Connectome for Long-Term Fear Memory in Mice
Source: PLoS Comput Biol. 2013 Jan 3;9(1):e1002853. doi: 10.1371/journal.pcbi.1002853 (PMC3536620; doi:10.1371/journal.pcbi.1002853)
Supplement: Table S5 — Brain regions implicated in long-term memory expression at remote time-points following training. A pubmed-based search was conducted to identify brain regions where targeted, loss-of-function manipulations affect expression of remote memory expression (see supplementary references [46]–[73] in Text S1). Behavioral paradigms were considered if acquisition and/or initial memory expression (e.g., 24 hours after training) were dependent upon the hippocampus. Loss-of-function manipulations included anatomical lesions, pharmacological inactivation, DNA demethylation, and testing occurred at least 25 days after the completion of training. DNMT = DNA methyltransferases. adorsal hippocampus, bventral hippocampus, cdorsal+ventral hippocampus. (PDF) [file pcbi.1002853.s020.pdf]

Region (s)	Manipulation	Behaviour	Reference	Retention delay (days)
Cg-a	inactivation	context fear conditioning	Frankland et al., 2004	36
Cg-a	inactivation	spatial memory (morris water maze)	Teixeira et al., 2006	30
Cg-a	inactivation	spatial memory (5 arm maze)	Maviel et al., 2004	30
Cg-a	inhibition of spine growth	context fear conditioning	Vetere et al., 2011	42
Cg-a	DNMT inhibition	context fear conditioning	Miller et al., 2010	30
mPFC (PrL & IL)	inactivation	trace fear conditioning	Blum et al., 2006	30
mPFC (PrL & IL)	inactivation	spatial memory (5 arm maze)	Maviel et al., 2004	30
mPFC (PrL & IL)	lesions	trace classical eyeblink conditioning	Takehara et al., 2003	32
mPFC (PrL & IL)	lesions	trace fear conditioning	Quinn et al., 2008	212
ILN/LT (LD, MD & C)	lesions	spatial memory (morris water maze)	Lopez et al., 2009	25
RSG	lesions	place discrimination (radial maze)	Haijima & Ichitani, 2008	35
RSG	inactivation	context fear conditioning	Corcoran et al., 2011	36
PRh	lesions	context fear conditioning	Burwell et al., 2004	>42
BL	lesions	context fear conditioning	Gale et al., 2004	502
Ce	lesions	fear-potentiated startle	Kim & Davis, 1993	37
OFC (MO, VO, LO, DLO)	inactivation	social transmission of food preference	Lesburguères et al., 2011	30
hippocampus [a]	inactivation	spatial memory (morris water maze)	Broadbent et al., 2006	30
hippocampus [a, c]	lesions	context fear conditioning	Lehmann et al., 2007	>104
hippocampus [a, b, c]	lesions	context fear conditioning	Sutherland et al., 2008	100
hippocampus [c]	lesions	fear potentiated startle	Lehmann et al., 2010	42
hippocampus [a, b, c]	lesions	morris water maze	Martin et al., 2005	56
hippocampus [c]	lesions	object fear conditioning	Lehmann et al., 2006	28
hippocampus [c]	lesions	picture discrimination	Epp et al., 2008	>37
hippocampus [c]	lesions	spatial memory (cross maze)	Winocur et al., 2005	>28
hippocampus [a, c]	lesions	spatial memory (water and land maze)	Clark et al., 2005	>77
hippocampus [a]	lesions	spatial reference memory (radial maze)	Ramos, 2009	82
hippocampus [c]	lesions	water maze (early life training)	Clark et al., 2005	114
hippocampus [c]	lesions	water maze (modified)	Clark et al., 2007	74
hippocampus [a]	lesions	place discrim (radial maze)	Haijima & Ichitani, 2008	35
