# Supplementary material for: Identification of a Functional Connectome for Long-Term Fear Memory in Mice
Source: PLoS Comput Biol. 2013 Jan 3;9(1):e1002853. doi: 10.1371/journal.pcbi.1002853 (PMC3536620; doi:10.1371/journal.pcbi.1002853)
Supplement: Text S1 — Supplementary methods, results, notes and references. (PDF) [file pcbi.1002853.s021.pdf]

Identification of a functional network for long-term fear memory in mice

Supplementary Text 1

Wheeler, Wang, Teixeira, Xiong, Kovacevic, Lerch, McIntosh, Parkinson & Frankland

Supplementary Methods

Relationship between magnitude and variance of regional Fos signal and correlation strength

We additionally evaluated the relationship between the magnitude and variance of our regional Fos signal and the tendency to see correlations with Fos expression in other brain regions. The mean and coefficient of variation (standard deviation/mean) were computed for the Fos counts in each of the 84 brain regions in the four groups of animals (WT/1 day, WT/36 days, α-CaMKII$^{+/-}$/1 day and α-CaMKII$^{+/-}$/36 days). The Pearson correlation coefficient was calculated to assess the association between (i) the mean Fos level and the mean squared Pearson correlation coefficient (mean r^2) and (ii) the coefficient of variation of the Fos signal and the mean r^2 by region across all 4 conditions.

Evaluation of reuniens thalamic nucleus anatomical connectivity

The direct anatomical connectivity of the reuniens thalamic nucleus was assessed by mining published data that investigated reuniens connectivity with infusion of tracers into the rodent brain. These studies were identified with the aid of an online connectivity database (The Brain Architecture Management System[1] [BAMS; [http://brancusi.usc.edu/bkms/]). Since the BAMS database remains sparsely populated, in addition this information was supplemented with studies identified through a PubMed search of the literature (http://www.ncbi.nlm.nih.gov/pubmed).

Context specificity of memory at short and long retention delays

In the main experiment, mice were trained and tested in context A. In order to assess whether the precision of the fear memory changes over time, we trained additional groups of mice using an identical protocol and then assessed their freezing in the training context (context A) and an alternate context (context B) either 1 or 36 days later. Context A was identical to the main experiment. For context B, a white plastic floor covered the shock grid bars, and a white, plastic, triangular insert was placed inside the conditioning chamber. As in the main experiment,

mice were handled prior to training (3 days, 2 min/day). One day following the completion of handling, mice were placed in context A for seven minutes. After two minutes they were presented with five unsignalled footshocks (2 s duration, 0.75 mA, 1 minute apart). Following the last footshock mice remained in the context for an additional minute, and were returned to their home cage. Either 1 day (n = 12) or 36 days (n = 14) later, freezing was assessed in contexts A and B. Tests were 3 min in duration, spaced ~5 h apart, and presented in a counter-balanced order. To compare discrimination across groups, we used the freezing scores to compute the following index: [freezing_A – freezing_B]/[freezing_A+ freezing_B].

Supplementary Results

Relationship between magnitude and variance of regional Fos signal and correlation strength

We found that functional connectivity was influenced both by retention delay and by genotype (Figure 2 and Figure 8). Importantly, these differences were independent of any group differences in Fos activation. For example, overall Fos levels were elevated in WT mice at the remote time-point (planned t-tests, WT/36 day > WT/1 days, α-CaMKII$^{+/-}$/1 day, α-CaMKII$^{+/-}$/36 days; *P*s < 0.05) (**Figure S4A**). However, regional correlation strength was not dependent upon Fos levels (or signal strength) (r = 0.021; *P* = 0.71) (**Figure S4B**), and therefore increased network connectivity in WT mice at the remote time-point is not simply a consequence of generally increased levels of activation. Moreover, while correlation strength typically increased as a function of variance (or coefficient of variation) (r = 0.198; *P* < 0.001), variance was equivalent across groups and therefore cannot account for increased network connectivity in WT mice at the remote time-point (planned t-tests, all comparisons *P*s > 0.05) (**Figure S4C-D**).

Patterns of inter-regional correlations derived from Fos and Egr-1 expression are similar

A number of other immediate early genes are regulated by neural activity, including *egr-1* [2]. In order to explore the generality of our effects we additionally quantified Egr-1 expression in a subset of brain regions in the WT/36 d group. We found that Fos- and Egr-1-derived patterns of inter-regional correlations were similar (**Figure S5**): Overall correlation strength did not differ in the Fos vs. Egr-1 matrices (by permutation testing; *P* = 0.76), nor were any individual inter-regional correlations different (*P* > 0.05 for all comparisons, corrected for multiple comparisons

using the False Discovery rate set at 5%). These results are consistent with previous studies showing that immediate early genes are typically expressed in the same or largely overlapping neuronal populations[3].

Time-dependent changes in context generalization

Mice were trained in context A and then tested in contexts A and B either 1 or 36 days later. At the short delay, mice froze more in context A compared to context B. In contrast, at the long delay, mice exhibited robust, but equivalent levels of freezing in either context (context × delay ANOVA; significant context × delay interaction; $F(1,54) = 7.89$, $P < 0.005$; planned paired t-tests indicated that freezing was greater in context A vs. B at the short [$t(11) = 4.30$, $P < 0.005$] but not long [$t(13) = 0.31$, $P > 0.05$] delay) (**Figure S14B**). Reflecting this time dependent increase in context generalization, discrimination declined as a function of retention delay (unpaired t-test, $t(24) = 3.37$, $P < 0.005$]) (**Figure S14C**). These time-dependent changes in context generalization are consistent with the idea that the contextual fear memory is transformed from a precise, detailed form into a less precise, generalized form[4].

Supplementary Notes

Defining functional connections on the basis of inter-regional analysis of Fos expression. In brain imaging studies, two regions are said to be functionally connected if their activity co-varies. Co-variance may be computed either within subjects (which is typically the case in human imaging studies) or between subjects (more typical in experimental animal studies where 'activity' is inferred post-mortem by changes in expression of activity-regulated genes, for example). Functional connections therefore reflect a statistical (rather than physical) relationship between two regions. As a purely statistical construct, functional connections may therefore be defined on multiple timescales, and the timescale depends on how 'activity' is being measured. For example, in electrophysiological studies, correlated patterns of spiking between two regions would define functional connections on the millisecond or second timescale. In contrast, correlated increases in the expression of an activity-regulated gene such as *c-fos* across two regions would define functional connections on the minutes to hours timescale, as Fos is induced by sustained neural activation and Fos expression peaks after 60-90 minutes.

Fos expression reflects sustained neural activation. To study the relationship between stimulation and Fos expression *in vivo*, we previously electrically stimulated of the entorhinal cortex, and examined Fos expression in granule cells in the dentate gyrus [3](see Figure 1). Importantly, we found that increases in Fos expression in dentate granule cells were 1) similar in magnitude to those following behavioral testing (e.g., placement of mouse in context previously paired with shock), 2) anatomically-specific (limited to dentate granule cells ipsilateral to stimulation site, consistent with predominantly unilateral efferent connections from the entorhinal cortex to the dentate gyrus) and 3) localized to the same subpopulations of granule cells expressing other activity-regulated genes (e.g., Arc). These *in vivo* data suggest that Fos induction reflects sustained neuronal activation.

Control networks. Since the emergence of applying graph theoretical approaches to study real life networks there has been much discussion and research into the choice of approaches for generating appropriate control networks (e.g.[5,6]). There are many ways in which random graphs may be constructed, and the choice of method will result in markedly different connection properties. For example, one could simply generate a network in which a set number of connections between nodes are randomly assigned. For the purposes of providing appropriate controls that reflect the connection distributions of the network being studied, a standard method (and the one we adopt here) is to shuffle the connections between nodes, while maintaining the same number of connections for each node [7]. This not only preserves the overall degree distribution of the network, but also ensures that each node has the same number of connections (albeit to different partners) as the original network. In this way we can examine how the global properties of the network vary from our so-called 'random networks' in which local properties of organization are preserved.

Supplementary References

1. Bota, M., Dong, H.W. & Swanson, L.W. Brain architecture management system. Neuroinformatics 3, 15-48 (2005).
2. Guzowski, J.F. et al. Mapping behaviorally relevant neural circuits with immediate-early gene expression. Curr Opin Neurobiol 15, 599-606 (2005).
3. Stone, S.S. et al. Functional convergence of developmentally and adult-generated granule cells in dentate gyrus circuits supporting hippocampus-dependent memory. Hippocampus 21, 1348-62 (2011).
4. Winocur, G., Moscovitch, M. & Sekeres, M. Memory consolidation or transformation: context manipulation and hippocampal representations of memory. Nat Neurosci 10, 555-7 (2007).
5. Barabasi, A.L. & Albert, R. Emergence of scaling in random networks. Science 286, 509-12 (1999).
6. Sales-Pardo, M., Guimera, R., Moreira, A.A. & Amaral, L.A. Extracting the hierarchical organization of complex systems. Proc Natl Acad Sci U S A 104, 15224-9 (2007).
7. Maslov, S. & Sneppen, K. Specificity and stability in topology of protein networks. Science 296, 910-3 (2002).
8. Franklin, K.B.J. & Paxinos, G. The mouse brain in stereotaxic coordinates, (Academic Press, San Diego, 2007).
9. Allen, G.V. & Cechetto, D.F. Functional and anatomical organization of cardiovascular pressor and depressor sites in the lateral hypothalamic area. II. Ascending projections. J Comp Neurol 330, 421-38 (1993).
10. Arnault, P. & Roger, M. Ventral temporal cortex in the rat: connections of secondary auditory areas Te2 and Te3. J Comp Neurol 302, 110-23 (1990).
11. Beckstead, R.M. An autoradiographic examination of corticocortical and subcortical projections of the mediodorsal-projection (prefrontal) cortex in the rat. J Comp Neurol 184, 43-62 (1979).
12. Beckstead, R.M., Domesick, V.B. & Nauta, W.J. Efferent connections of the substantia nigra and ventral tegmental area in the rat. Brain Res 175, 191-217 (1979).
13. Canteras, N.S. & Swanson, L.W. Projections of the ventral subiculum to the amygdala, septum, and hypothalamus: a PHAL anterograde tract-tracing study in the rat. J Comp Neurol 324, 180-94 (1992).
14. Canteras, N.S., Simerly, R.B. & Swanson, L.W. Organization of projections from the ventromedial nucleus of the hypothalamus: a Phaseolus vulgaris-leucoagglutinin study in the rat. J Comp Neurol 348, 41-79 (1994).
15. Cenquizca, L.A. & Swanson, L.W. Analysis of direct hippocampal cortical field CA1 axonal projections to diencephalon in the rat. J Comp Neurol 497, 101-14 (2006).
16. Deacon, T.W., Eichenbaum, H., Rosenberg, P. & Eckmann, K.W. Afferent connections of the perirhinal cortex in the rat. J Comp Neurol 220, 168-90 (1983).
17. Dong, H.W. & Swanson, L.W. Projections from bed nuclei of the stria terminalis, posterior division: implications for cerebral hemisphere regulation of defensive and reproductive behaviors. J Comp Neurol 471, 396-433 (2004).
18. Dong, H.W. & Swanson, L.W. Organization of axonal projections from the anterolateral area of the bed nuclei of the stria terminalis. J Comp Neurol 468, 277-98 (2004).

19. Dong, H.W. & Swanson, L.W. Projections from bed nuclei of the stria terminalis, magnocellular nucleus: implications for cerebral hemisphere regulation of micturition, defecation, and penile erection. J Comp Neurol 494, 108-41 (2006).
20. Dong, H.W. & Swanson, L.W. Projections from bed nuclei of the stria terminalis, dorsomedial nucleus: implications for cerebral hemisphere integration of neuroendocrine, autonomic, and drinking responses. J Comp Neurol 494, 75-107 (2006).
21. Dong, H.W. & Swanson, L.W. Projections from bed nuclei of the stria terminalis, anteromedial area: cerebral hemisphere integration of neuroendocrine, autonomic, and behavioral aspects of energy balance. J Comp Neurol 494, 142-78 (2006).
22. Dong, H.W., Petrovich, G.D., Watts, A.G. & Swanson, L.W. Basic organization of projections from the oval and fusiform nuclei of the bed nuclei of the stria terminalis in adult rat brain. J Comp Neurol 436, 430-55 (2001).
23. Dreher, B., Dehay, C. & Bullier, J. Bihemispheric Collateralization of the Cortical and Subcortical Afferents to the Rat's Visual Cortex. Eur J Neurosci 2, 317-331 (1990).
24. Finch, D.M., Derian, E.L. & Babb, T.L. Afferent fibers to rat cingulate cortex. Exp Neurol 83, 468-85 (1984).
25. Freedman, L.J. & Cassell, M.D. Thalamic afferents of the rat infralimbic and lateral agranular cortices. Brain Res Bull 26, 957-64 (1991).
26. Goto, M., Canteras, N.S., Burns, G. & Swanson, L.W. Projections from the subfornical region of the lateral hypothalamic area. J Comp Neurol 493, 412-38 (2005).
27. van Groen, T. & Wyss, J.M. Connections of the retrosplenial granular a cortex in the rat. J Comp Neurol 300, 593-606 (1990).
28. Hurley, K.M., Herbert, H., Moga, M.M. & Saper, C.B. Efferent projections of the infralimbic cortex of the rat. J Comp Neurol 308, 249-76 (1991).
29. McKenna, J.T. & Vertes, R.P. Afferent projections to nucleus reuniens of the thalamus. J Comp Neurol 480, 115-42 (2004).
30. Petrovich, G.D., Risold, P.Y. & Swanson, L.W. Organization of projections from the basomedial nucleus of the amygdala: a PHAL study in the rat. J Comp Neurol 374, 387-420 (1996).
31. Risold, P.Y. & Swanson, L.W. Connections of the rat lateral septal complex. Brain Res Brain Res Rev 24, 115-95 (1997).
32. Risold, P.Y. & Swanson, L.W. Evidence for a hypothalamothalamocortical circuit mediating pheromonal influences on eye and head movements. Proc Natl Acad Sci U S A 92, 3898-902 (1995).
33. Risold, P.Y., Canteras, N.S. & Swanson, L.W. Organization of projections from the anterior hypothalamic nucleus: a Phaseolus vulgaris-leucoagglutinin study in the rat. J Comp Neurol 348, 1-40 (1994).
34. Risold, P.Y., Thompson, R.H. & Swanson, L.W. The structural organization of connections between hypothalamus and cerebral cortex. Brain Res Brain Res Rev 24, 197-254 (1997).
35. Sesack, S.R., Deutch, A.Y., Roth, R.H. & Bunney, B.S. Topographical organization of the efferent projections of the medial prefrontal cortex in the rat: an anterograde tract-tracing study with Phaseolus vulgaris leucoagglutinin. J Comp Neurol 290, 213-42 (1989).
36. Shin, J.W., Geerling, J.C. & Loewy, A.D. Inputs to the ventrolateral bed nucleus of the stria terminalis. J Comp Neurol 511, 628-57 (2008).

37. Simerly, R.B. & Swanson, L.W. Projections of the medial preoptic nucleus: a Phaseolus vulgaris leucoagglutinin anterograde tract-tracing study in the rat. J Comp Neurol 270, 209-42 (1988).
38. Sripanidkulchai, K. & Wyss, J.M. Thalamic projections to retrosplenial cortex in the rat. J Comp Neurol 254, 143-65 (1986).
39. Thompson, R.H., Canteras, N.S. & Swanson, L.W. Organization of projections from the dorsomedial nucleus of the hypothalamus: a PHA-L study in the rat. J Comp Neurol 376, 143-73 (1996).
40. Thompson, R.H. & Swanson, L.W. Structural characterization of a hypothalamic visceromotor pattern generator network. Brain Res Brain Res Rev 41, 153-202 (2003).
41. Thompson, S.M. & Robertson, R.T. Organization of subcortical pathways for sensory projections to the limbic cortex. I. Subcortical projections to the medial limbic cortex in the rat. J Comp Neurol 265, 175-88 (1987).
42. Vertes, R.P., Hoover, W.B., Do Valle, A.C., Sherman, A. & Rodriguez, J.J. Efferent projections of reuniens and rhomboid nuclei of the thalamus in the rat. J Comp Neurol 499, 768-96 (2006).
43. Woolf, N.J., Eckenstein, F. & Butcher, L.L. Cholinergic systems in the rat brain: I. projections to the limbic telencephalon. Brain Res Bull 13, 751-84 (1984).
44. Wyss, J.M., Swanson, L.W. & Cowan, W.M. A study of subcortical afferents to the hippocampal formation in the rat. Neuroscience 4, 463-76 (1979).
45. Zeng, D. & Stuesse, S.L. Morphological heterogeneity within the cingulate cortex in rat: a horseradish peroxidase transport study. Brain Res 565, 290-300 (1991).
46. Broadbent, N.J., Squire, L.R. & Clark, R.E. Reversible hippocampal lesions disrupt water maze performance during both recent and remote memory tests. Learn Mem 13, 187-91 (2006).
47. Clark, R.E., Broadbent, N.J. & Squire, L.R. Impaired remote spatial memory after hippocampal lesions despite extensive training beginning early in life. Hippocampus 15, 340-6 (2005).
48. Clark, R.E., Broadbent, N.J. & Squire, L.R. Hippocampus and remote spatial memory in rats. Hippocampus 15, 260-72 (2005).
49. Clark, R.E., Broadbent, N.J. & Squire, L.R. The hippocampus and spatial memory: findings with a novel modification of the water maze. J Neurosci 27, 6647-54 (2007).
50. Epp, J. et al. Retrograde amnesia for visual memories after hippocampal damage in rats. Learn Mem 15, 214-21 (2008).
51. Lehmann, H., Lacanilao, S. & Sutherland, R.J. Complete or partial hippocampal damage produces equivalent retrograde amnesia for remote contextual fear memories. Eur J Neurosci 25, 1278-86 (2007).
52. Lehmann, H., Lecluse, V., Houle, A. & Mumby, D.G. Retrograde amnesia following hippocampal lesions in the shock-probe conditioning test. Hippocampus 16, 379-87 (2006).
53. Lehmann, H., Sparks, F.T., O'Brien, J., McDonald, R.J. & Sutherland, R.J. Retrograde amnesia for fear-potentiated startle in rats after complete, but not partial, hippocampal damage. Neuroscience 167, 974-84 (2010).
54. Martin, S.J., de Hoz, L. & Morris, R.G. Retrograde amnesia: neither partial nor complete hippocampal lesions in rats result in preferential sparing of remote spatial memory, even after reminding. Neuropsychologia 43, 609-24 (2005).

55. Ramos, J.M. Remote spatial memory and the hippocampus: effect of early and extensive training in the radial maze. Learn Mem 16, 554-63 (2009).
56. Sutherland, R.J., O'Brien, J. & Lehmann, H. Absence of systems consolidation of fear memories after dorsal, ventral, or complete hippocampal damage. Hippocampus 18, 710-8 (2008).
57. Winocur, G., Moscovitch, M., Caruana, D.A. & Binns, M.A. Retrograde amnesia in rats with lesions to the hippocampus on a test of spatial memory. Neuropsychologia 43, 1580-90 (2005).
58. Blum, S., Hebert, A.E. & Dash, P.K. A role for the prefrontal cortex in recall of recent and remote memories. Neuroreport 17, 341-4 (2006).
59. Burwell, R.D., Bucci, D.J., Sanborn, M.R. & Jutras, M.J. Perirhinal and postrhinal contributions to remote memory for context. J Neurosci 24, 11023-8 (2004).
60. Corcoran, K.A. et al. NMDA Receptors in Retrosplenial Cortex Are Necessary for Retrieval of Recent and Remote Context Fear Memory. J Neurosci 31, 11655-9 (2011).
61. Frankland, P.W., Bontempi, B., Talton, L.E., Kaczmarek, L. & Silva, A.J. The involvement of the anterior cingulate cortex in remote contextual fear memory. Science 304, 881-3 (2004).
62. Gale, G.D. et al. Role of the basolateral amygdala in the storage of fear memories across the adult lifetime of rats. J Neurosci 24, 3810-5 (2004).
63. Haijima, A. & Ichitani, Y. Anterograde and retrograde amnesia of place discrimination in retrosplenial cortex and hippocampal lesioned rats. Learn Mem 15, 477-82 (2008).
64. Kim, M. & Davis, M. Lack of a temporal gradient of retrograde amnesia in rats with amygdala lesions assessed with the fear-potentiated startle paradigm. Behav Neurosci 107, 1088-92 (1993).
65. Lesburgueres, E. et al. Early tagging of cortical networks is required for the formation of enduring associative memory. Science 331, 924-8 (2011).
66. Lopez, J. et al. The intralaminar thalamic nuclei contribute to remote spatial memory. J Neurosci 29, 3302-6 (2009).
67. Maviel, T., Durkin, T.P., Menzaghi, F. & Bontempi, B. Sites of neocortical reorganization critical for remote spatial memory. Science 305, 96-9 (2004).
68. Miller, C.A. et al. Cortical DNA methylation maintains remote memory. Nat Neurosci 13, 664-6 (2010).
69. Quinn, J.J., Ma, Q.D., Tinsley, M.R., Koch, C. & Fanselow, M.S. Inverse temporal contributions of the dorsal hippocampus and medial prefrontal cortex to the expression of long-term fear memories. Learn Mem 15, 368-72 (2008).
70. Takehara, K., Kawahara, S. & Kirino, Y. Time-dependent reorganization of the brain components underlying memory retention in trace eyeblink conditioning. J Neurosci 23, 9897-905 (2003).
71. Teixeira, C.M., Pomedli, S.R., Maei, H.R., Kee, N. & Frankland, P.W. Involvement of the anterior cingulate cortex in the expression of remote spatial memory. J Neurosci 26, 7555-64 (2006).
72. Vetere, G. et al. Spine growth in the anterior cingulate cortex is necessary for the consolidation of contextual fear memory. Proc Natl Acad Sci U S A 108, 8456-60 (2011).
73. Goshen, I. et al. Dynamics of retrieval strategies for remote memories. Cell 147, 678-89 (2011).
